# In Situ Generated Sulfate-Facilitated Efficient Nitrate Electrosynthesis on 2D PdS_2_ with Unique Imitating Growth Feature

**DOI:** 10.1007/s40820-025-01803-3

**Published:** 2025-06-12

**Authors:** Rui Zhang, Hui Mao, Ziyi Wang, Shengke Ma, Shuyao Wu, Qiong Wu, Daliang Liu, Hui Li, Yang Fu, Xiaoning Li, Tianyi Ma

**Affiliations:** 1 Shenyang Key Laboratory of Medical Molecular Theranostic Probes in School of Pharmacy, School of Pharmacy, Shenyang Medical College, Shenyang, 110034 People’s Republic of China; 2 Centre for Atomaterials and Nanomanufacturing (CAN), School of Science, RMIT University, Melbourne, VIC 3000 Australia; 3 Institute of Clean Energy Chemistry, Liaoning Key Lab for Green Synthesis and Preparative Chemistry of Advanced Materials, College of Chemistry, Liaoning University, Shenyang, 110036 People’s Republic of China; 4 ARC Industrial Transformation Research Hub for Intelligent Energy Efficiency in Future Protected Cropping (E2Crop), Melbourne, VIC 3000 Australia

**Keywords:** Imitating growth, PdS_2_, Nanoplates, Nitrogen oxidation reaction (NOR), Synergistic effect

## Abstract

As a green sustainable alternative technology, synthesizing nitrate by electrocatalytic nitrogen oxidation reaction (NOR) can replace the traditional energy-intensive Ostwald process. But low nitrogen fixation yields and poor selectivity due to the high bond energy of the N≡N bond and competition from the oxygen evolution reaction in the electrolyte restrict its application. On the other hand, two-dimensional (2D) PdS_2_ as a member in the family of group-10 novel transition metal dichalcogenides (NTMDs) presents the interesting optical and electronic properties due to its novel folded pentagonal structure, but few researches involve to its fabrication and application. Herein, unique imitating growth feature for PdS_2_ on different 2D substrates has been firstly discovered for constructing 2D/2D heterostructures by interface engineering. Due to the different exposed chemical groups on the substrates, PdS_2_ grows as the imitation to the morphologies of the substrates and presents different thickness, size, shape and the degree of oxidation, resulting in the significant difference in the NOR activity and stability of the obtained composite catalysts. Especially, the thin and small PdS_2_ nanoplates with more defects can be obtained by decorating poly(1-vinyl-3-ethylimidazolium bromide) on the 2D substrate, easily oxidized during the preparation process, resulting in the in situ generation of SO_4_^2−^, which plays a crucial role in reducing the activation energy of the NOR process, leading to improved efficiency for nitrate production, verified by theoretical calculation. This research provides valuable insights for the development of novel electrocatalysts based on NTMDs for NOR and highlights the importance of interface engineering in enhancing catalytic performance.

**Supplementary Information:**

The online version contains supplementary material available at 10.1007/s40820-025-01803-3.

## Highlights

- Unique imitating growth feature for PdS_2_ on different 2D substrates has been firstly discovered for constructing 2D/2D heterostructures by interface engineering.
- The thin and small PdS_2_ nanoplates with active defects can be inducted by poly(1-vinyl-3-ethylimidazolium bromide (PVEIB), resulting in the obtained PdS_2_@PVEIB/PPy/GO exhibited the excellent nitrogen oxidation reaction (NOR) electroactivity with the outstanding stability and selectivity.
- The in situ generation of SO_4_^2−^, caused by the oxidation during the preparation process or exposed in air, as well as at high NOR potential, plays a crucial role in reducing the activation energy of the NOR process, leading to improved efficiency for nitrate production.

**Supplementary Information:**

The online version contains supplementary material available at 10.1007/s40820-025-01803-3.

## Graphical abstract

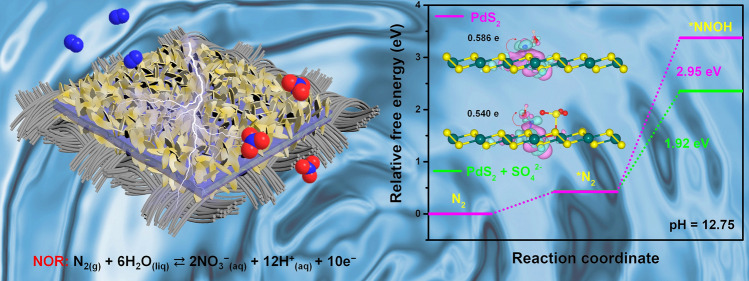

## Introduction

With the increase in pressure of energy crisis and environmental pollution, more and more attentions have been attracted to the development of more sustainable alternatives to replace the traditional technologies based on renewable energy and green energy conversion technology [1–3]. Electrocatalysis is considered one of the most effective approaches to alleviate energy problems by using renewable energy-generated electricity to sustainably fuels or value-added chemicals [4–7]. Exploring stable and efficient electrocatalysts is the key to improve the rate of electrocatalysis, which has important strategic significance for the development of advanced energy conversion devices. Recently, electrocatalytic nitrogen oxidation reaction (NOR) has received extensive attentions, which involves the oxidation of N_2_ on the electrode surface to produce nitrates or other nitrogen oxides; therefore, it is expected to become a green sustainable alternative technology for synthesizing nitrate under mild conditions, replacing the traditional Ostwald process with high energy consumption and large amounts of carbon dioxide emission [8–10]. The selection and design of the electrocatalysts are essential to NOR, because the activity, stability and selectivity of the catalyst can directly affect the efficiency of NOR and the quality of the product. Therefore, many attentions have been focused on developing efficient, stable and sustainable electrocatalysts to improve the NOR performance [11–13].

Recently, a breakthrough of improved NOR electroactivity has been achieved in FeS_2_-TiO_2_ heterogeneous nanoparticles or Pd^2+^/S^2−^-doped TiO_2_ nanoparticles supported on 2-methylimidazolium functionalized polypyrrole/graphene oxide [14, 15]. Although the exact mechanism is not fully understood, it is believed that in these S-containing materials, S^2−^ can be oxidized to persulfate reactive oxygen species (*SO_4_) by *O generated during the competitive oxygen evolution reaction (OER) at high potentials, which then cooperatively activate nitrogen and further accelerate NOR [16]. Predictably, the improving NOR electroactivity would be potentially achieved by developing S-containing NOR electrocatalysts with preexisting *SO_4_. Two-dimensional (2D) transition metal dichalcogenides (TMDs) possess unique electronic structure, large specific surface area and abundant surface/edge atoms, which can act as the promising electrocatalysts widely applied to water splitting and nitrogen fixation [17–19]. Many effective electrocatalysts based on 2D TMDs have been prepared for producing ammonia under ambient conditions by nitrogen reduction reaction (NRR); especially, the defect-rich 2D/2D heterostructures constructed by interface engineering exhibit improving catalytic activity and stability, because the crystal structure of 2D TMDs as the catalytic centers, such as MoS_2_, can be adjusted and controlled by the specific inducing groups on the interface, generating the more defects exposed as the active sites [20–22]. Due to the existence of the defects, the surface/edge S^2−^ can be also easily converted to SO_4_^2−^ by the oxidation, even during the synthesized process or exposed in the air, resulting in the preexistence of SO_4_^2−^ in 2D TMDs. Therefore, 2D TMDs are expected as the ideal electrocatalysts for NOR in alkaline system, balancing NOR and OER to achieve optimal NO_3_^−^ yield and Faraday efficiency (FE), even the excellent stability.

Different from the hexagonal structure in traditional TMDs, such as MoS_2_ and WS_2_, PdS_2_ as a member in the family of group-10 novel TMDs (NTMDs) possesses a novel folded pentagonal structure, resulting in the interesting optical and electronic properties [23–25]. Up to now, the reports related to PdS_2_ mostly have focused on theoretical calculations [26–29], and few literatures involve to the fabrication and application of PdS_2_, though PdS_2_ exhibited high electroactivity in hydrogen evolution reactions (HER) process [30] and fuel cells [31]. Herein, unique imitating growth feature for 2D PdS_2_ thin nanoplates on different 2D substrates has been firstly discovered and its electrocatalytic NOR performance can be significantly improved by the in situ generating *SO_4_ from the oxidation of surface/edge S^2−^ due to the existence of active defects. Specifically, PdS_2_ nanoplates can be anchored on the surfaces of graphene oxide (GO), polypyrrole/graphene oxide (PPy/GO) and poly(1-vinyl-3-ethylimidazolium bromide) functionalized polypyrrole/graphene oxide (PVEIB/PPy/GO) for constructing 2D/2D heterostructures by interface engineering. Caused by the different induction of the exposed chemical groups on the substrates, PdS_2_ grows as the imitation to the morphologies of the substrates and present different thickness, size, shape and the degree of oxidation, resulting in the significant difference in the electrocatalytic NOR activity and stability of the obtained composite catalysts. The most excellent NOR performance with the significantly promoted stability can be achieved by PdS_2_@PVEIB/PPy/GO among the obtained three 2D/2D heterostructures due to the synergistic effect of each component. Especially, the thin and small PdS_2_ nanoplates with more defects can be obtained by the induction of PVEIB, easily oxidized during the preparation process or exposed in air, resulting in the in situ generation of SO_4_^2−^, which plays a crucial role in reducing the activation energy of the NOR process, leading to improved efficiency for nitrate production, verified by both experimental and theoretical evidence. This research provides valuable insights for the development of novel electrocatalysts based on NTMDs for NOR and highlights the importance of interface engineering in enhancing catalytic performance.

## Experimental Section

### Preparation of Electrocatalysts Based on PdS_2_

PdS_2_ nanoplates were prepared by a simple hydrothermal process. In a typical experiment, K_2_PdCl_6_ (4.24 mL, 19.1 mg mL^−1^) and thioacetamide (TAA, 113.4 mg) were dissolved in another 46 mL ultra-pure water, stirring for 10 min. Then, the mixture was transferred and sealed into a 100 mL of Teflon-lined stainless steel autoclave and maintained at 200 °C for 24 h. Finally, the dark gray powders were collected and washed with ethanol and water by centrifugation and then dried in vacuum at 50 °C for 24 h. PdS_2_@GO, PdS_2_@PPy/GO and PdS_2_@PVEIB/PPy/GO were also prepared by adjusting the content of precursor under the same conditions with different substrates. The used chemical reagents, preparation methods of the substrates, characterizations and apparatus were shown in the Supporting Information in detail.

### Preparation of the Working Electrode for NOR

Separately selecting the pure PdS_2_ nanoplates, PdS_2_@GO, PdS_2_@PPy/GO and PdS_2_@PVEIB/PPy/GO as the electrocatalysts, the electrocatalysts suspension was prepared by adding 1.5 mg of electrocatalysts into 460 μL ethanol mixed with 40 μL Nafion perfluorinated resin solution, and ultrasound dispersed evenly for 30 min. A working electrode was constructed by dropping the above catalyst suspension onto a carbon cloth (CC) with area of 1.0 cm × 1.0 cm, used for conducting chronoamperometric measurement. The mass of electrocatalysts coating on CC was calculated by weighing after dried at 50 °C for 12 h.

### Electrochemical Measurements and Nitrate Detection

The electrochemical performance of electrocatalysts based on PdS_2_ was investigated by chronoamperometry with a CHI1040C Electrochemical Station (Shanghai CHENHUA Instrument Co., Ltd.). All the measurements were performed in 0.1 M KOH (pH = 12.75) containing saturated Ar or N_2_ at room temperature. The chronoamperometry tests for detecting NO_3_^−^ were performed in 50 mL of 0.1 M KOH solution in a standard three-electrode system using electrocatalysts coated on CC as the working electrode, a platinum plate as the counter electrode and a Hg/HgO electrode as the reference electrode. The measured potentials versus Hg/HgO were converted to reversible hydrogen electrode (RHE) scale according to the Nernst equation (*E*_RHE_ = *E*_Hg/HgO_ + 0.059pH + 0.098).

The produced NO_3_^−^ was determined by an ion chromatography (IC). The standard curves and the calculation processes were shown in the Supporting Information in detail.

### Electrochemical In Situ ATR-SEIRAS and Computational Method

The NOR mechanism was deduced through density functional theory (DFT) calculations, based on these detected N-containing oxide species measured by electrochemical in situ attenuated total reflection surface-enhanced infrared absorption spectroscopy (ATR-SEIRAS). The parameters of both electrochemical in situ ATR-SEIRAS and DFT calculations were shown in the Supporting Information in detail.

## Results and Discussion

### Structural Characterization and NOR Electroactivity of PdS_2_

**Fig. 1:**
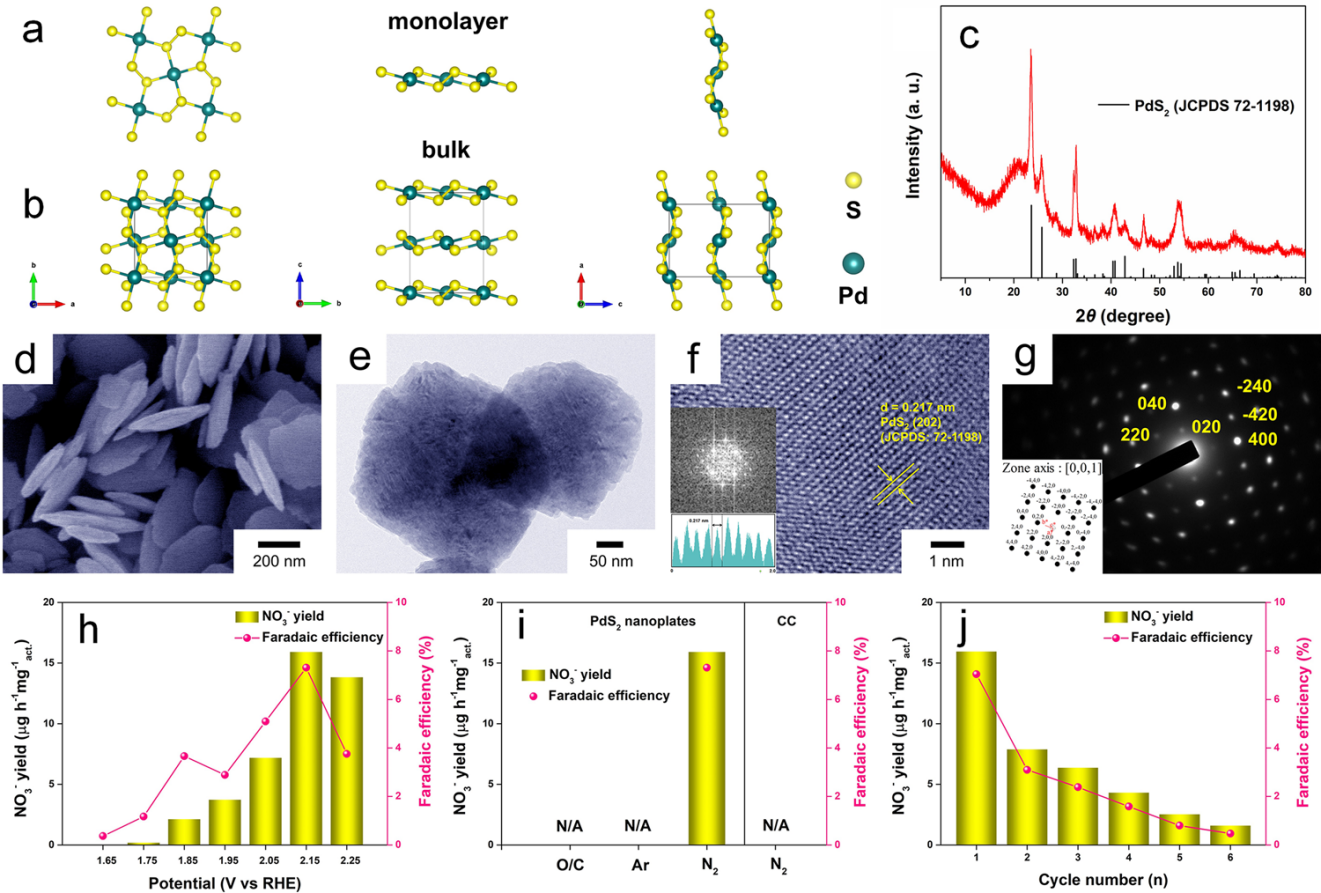
Top and side views of the pentagonal PdS_2_ **a** monolayer and **b** bulk; **c** XRD pattern, **d** SEM, **e** TEM, **f** HRTEM image with FFT pattern and **g** SAED pattern with the corresponding simulated diffraction pattern of PdS_2_ nanoplates; **h** NO_3_^−^ yield and FE of PdS_2_ nanoplates for NOR at different potentials (vs. RHE); **i** electroactivity of CC (substrate) and PdS_2_ nanoplates toward NOR at 2.15 V under different conditions; **j** cyclic test for PdS_2_ nanoplates performed six times at 2.15 V under electrolysis for 2 h

The geometric structure of pentagonal PdS_2_ is composed of three-atom-thick layers arranged in an S–Pd–S configuration, where a Pd layer is encapsulated between two S layers to form a sandwiched-like structure [28], and its monolayer structure and bulk are as illustrated in Fig. 1a, b, respectively. The synthesized PdS_2_ exhibits good crystallinity, as confirmed by their X-ray diffraction (XRD) pattern (Fig. 1c) showing several strong diffraction peaks that correspond well to the JCPDS No.72–1198. By scanning electron microscopy (SEM) and transmission electron microscopy (TEM), it can be clearly observed that the synthesized PdS_2_ has a quadrilateral nanoplate structure in Fig. 1d, e, whose thickness is about 75 nm. A lattice spacing of 0.217 nm measured in the high-resolution TEM (HRTEM) image (Fig. 1f) is well matched to the (202) lattice plane of orthorhombic PdS_2_, which is consistent with result of the lattice fringe measurement by the simulated fast Fourier transform (FFT) and the corresponding FFT pattern reveals that the orthorhombic PdS_2_ was monocrystalline. Especially, the characteristic diffraction spot pattern (Fig. 1g) obtained from selected area electron diffraction (SAED) aligns with orthorhombic PdS_2_ (JCPDS No. 72–1198), as well as the corresponding simulated diffraction pattern (Fig. 1g, inset), conclusively confirming the monocrystalline nature of the synthesized PdS_2_ nanoplates. Figure 1h displays the electrocatalytic NOR performance of PdS_2_ nanoplates at different potentials obtained by chronoamperometry (CA) test, with the NO_3_^−^ yield and FE as indicators, which are determined by an IC and calculated by a standard curve of NO_3_^−^ in Fig. S1. It is found that 2.15 V (vs. RHE) is the optimum potential of NOR electrocatalyzed by PdS_2_ nanoplates, with the highest NO_3_^−^ yield of 15.94 μg h^−1^ mg^−1^_act._ and the maximum FE of 7.31%. Obviously, the generated nitrate is exclusively derived from N_2_, because that no nitrate can be detected in the electrolyte after electrocatalyzed by CC under N_2_ atmosphere or by PdS_2_ under Ar atmosphere at 2.15 V, even under N_2_ atmosphere without external voltage in Fig. 1i. However, the practical application of PdS_2_ nanoplates for nitrate electrosynthesis is still hindered by the poor cycling performance (Fig. 1j).

### Improved NOR Electroactivity of PdS_2_ with Unique Imitating Growth Feature by Interface Engineering

**Fig. 2:**
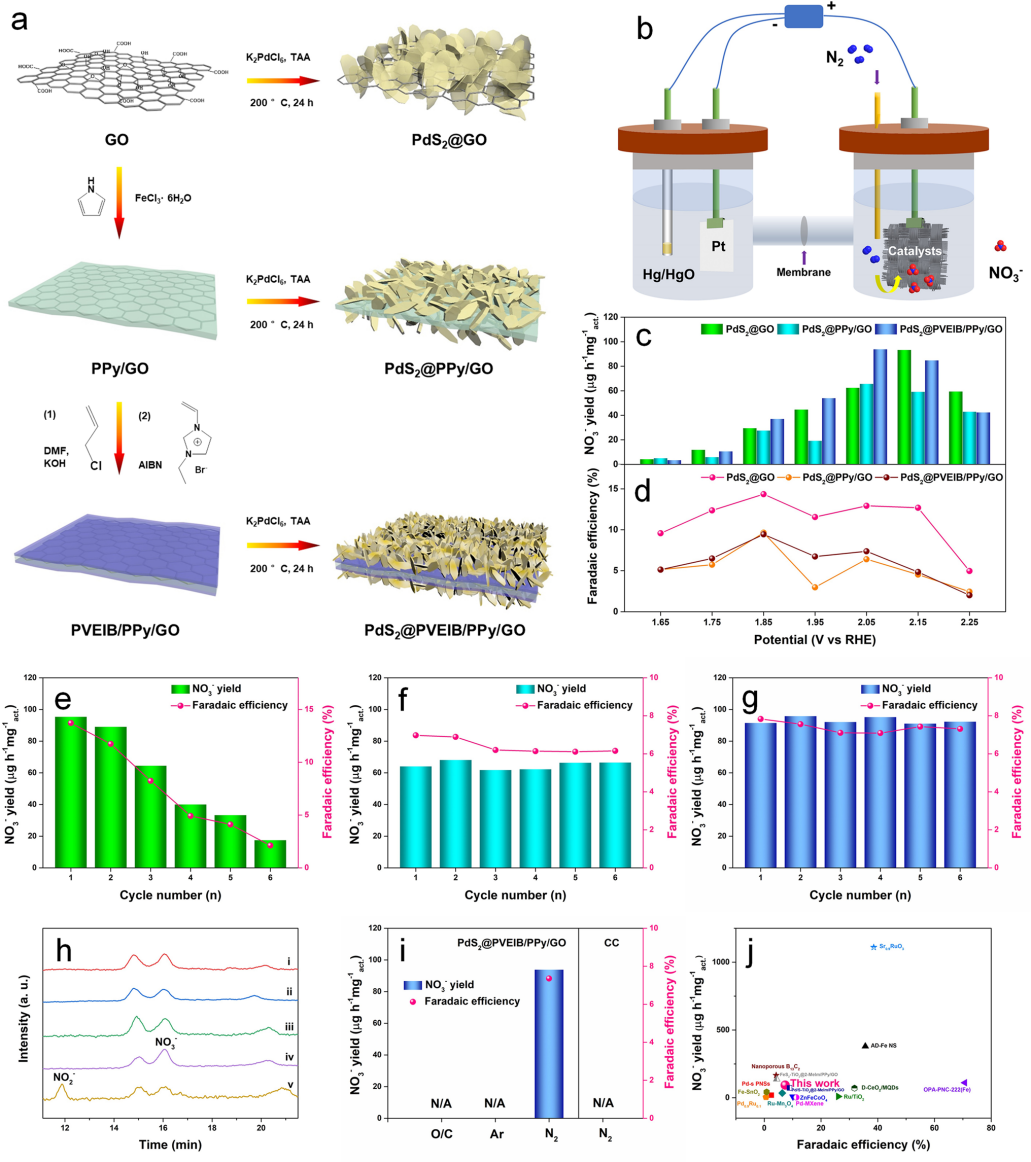
**a** Schematic of the synthetic process of PdS_2_@GO, PdS_2_@PPy/GO and PdS_2_@PVEIB/PPy/GO; **b** Schematic of an H-type cell for NOR; **c** NO_3_^−^ yield and **d** FE of PdS_2_@GO, PdS_2_@PPy/GO and PdS_2_@PVEIB/PPy/GO for NOR at different potentials (vs. RHE); cyclic test for **e** PdS_2_@GO at 2.15 V, **f** PdS_2_@PPy/GO and **g** PdS_2_@PVEIB/PPy/GO at 2.05 V under electrolysis for 2 h performed six times; **h** Ion chromatograms of the diluted electrolyte after NOR by i) PdS_2_@GO, ii) PdS_2_@PPy/GO and iii) PdS_2_@PVEIB/PPy/GO at 2.05 V, iv) the standard NO_3_^−^ of 0.2 μg mL^−1^, v) the standard NO_2_^−^ of 0.2 μg mL^−1^; **i** Electroactivity of CC (substrate) and PdS_2_@PVEIB/PPy/GO toward NOR at 2.05 V under different conditions; **j** Comparison of NOR electrocatalytic performance with some reported catalysts (see detailed information in Table S2)

As reported, the electrocatalytic activity and stability can be effectively enhanced by interface engineering [32], which is attributed to the distinct phase properties at the interface, directly impacting the adsorption energy and electron transport kinetics of intermediate products on either side of the electrocatalytic reaction interface [33]. Building 2D/2D heterostructures is a popular approach for interface engineering, which can availably improve the nitrogen fixation performance of electrocatalysts [21, 34]. Through adjusting the contents of Pd precursor, sulfur source and substrate, PdS_2_ nanoplates can be anchored on the surface of the pre-prepared GO, PPy/GO and PVEIB/PPy/GO for constructing typical 2D/2D heterostructures to improve the NOR electroactivity, as illustrated in Fig. 2a. The morphologies of the three substrates are displayed in Fig. S3. The linear sweep voltammetry (LSV) curves of all the electrocatalysts based on PdS_2_ in Fig. S4 present that the current density (*j*) obtained in N_2_-saturated electrolyte is higher than that obtained in Ar-saturated electrolyte, indicating the occurrence of NOR process catalyzed by these electrocatalysts. Obviously in Fig. 2c, d, the NO_3_^−^ yield detected by ion chromatography and the corresponding FE can be significantly promoted due to the electrocatalysis of PdS_2_@GO, PdS_2_@PPy/GO and PdS_2_@PVEIB/PPy/GO in the potential range of 1.65–2.35 V (vs. RHE), generated by electrocatalytic NOR in an H-type cell (Fig. 2b) and the corresponding chronoamperometry curves are shown in Fig. S5. Table S1 lists the Pd content determined by an inductively coupled plasma-optical emission spectrometry (ICP-OES) and the corresponding calculated PdS_2_ loading content of the three electrocatalysts. The optimum potential for electrocatalytic NOR obtained by the pure PdS_2_ nanoplates and PdS_2_@GO is 2.15 V (vs. RHE), but for PdS_2_@PPy/GO and PdS_2_@PVEIB/PPy/GO is 2.05 V (vs. RHE), indicating that the existence of PPy with good conductivity is conducive to the electron transport during the NOR process, resulting in reducing the NOR overpotential of the composite catalysts. The highest NO_3_^−^ yield reaches to 93.91 μg h^−1^ mg^−1^_act._ obtained by PdS_2_@PVEIB/PPy/GO at 2.05 V, with the corresponding FE of 7.36%. Though PdS_2_@GO presents the highest FE at each potential among the three catalysts (Fig. 2d), with the maximum NO_3_^−^ yield of 93.39 μg h^−1^ mg^−1^_act._ at 2.15 V (Fig. 2c) comparable to that of PdS_2_@PVEIB/PPy/GO, the cycle stability of PdS_2_@GO for NOR is very poor, which is similar to the pure PdS_2_ nanoplates. The NO_3_^−^ yield and FE obtained by PdS_2_@GO at 2.15 V rapidly decrease during six cycles, left to 18.4% of the initial value (Fig. 2e), while PdS_2_@PPy/GO exhibits the improved NOR stability with only a little decrease of the NO_3_^−^ yield and FE obtained at 2.05 V after six cycles (Fig. 2f). Especially, PdS_2_@PVEIB/PPy/GO exhibits the excellent NOR stability with only a slight fluctuation of both NO_3_^−^ yield and FE at 2.05 V for six cycles in Fig. 2g. Obviously, the excellent NOR stability obtained by PdS_2_@PPy/GO and PdS_2_@PVEIB/PPy/GO is attributed to the contribution of PPy with good conductivity, facilitating the electron conduction and avoiding the charge accumulation during the NOR process, which results in the continuous occurrence of NOR on the electrocatalysts. Analysis of the ion chromatograms of standard NO_3_^−^ and NO_2_^−^ in Fig. 2h reveals the detection of only NO_3_^−^ in N_2_-saturated electrolyte when electrocatalyzed by PdS_2_@GO, PdS_2_@PPy/GO and PdS_2_@PVEIB/PPy/GO, indicating the perfect selectivity of the 2D/2D heterostructures based on PdS_2_ for NOR in the nitrate electrosynthesis. In addition, no nitrate can be detected in the electrolyte after electrocatalyzed by CC under N_2_ atmosphere or by PdS_2_@PVEIB/PPy/GO under Ar atmosphere at 2.05 V, as well as under N_2_ atmosphere without external voltage in Fig. 2i, suggesting that the generated nitrate is exclusively derived from N_2_ through the electrocatalytic action of PdS_2_@PVEIB/PPy/GO. Compared to the reported NOR electrocatalysts listed in Table S2, Fig. 2j presents the NOR electroactivity of PdS_2_@PVEIB/PPy/GO is at the forefront, contributed by the synergistic effect of each component.

For further exploring why PdS_2_@PVEIB/PPy/GO exhibits the excellent electrocatalytic NOR performance, the morphologies and structures of PdS_2_@GO, PdS_2_@PPy/GO and PdS_2_@PVEIB/PPy/GO, as well as PdS_2_@PVEIB/PPy/GO after long term NOR, are thoroughly characterized by using SEM, HRTEM, XRD and X-ray photoelectron spectroscopy (XPS) method. All of the electrocatalysts as synthesized present typical 2D/2D heterostructures, but interestingly, significant differences are evident in the morphology of PdS_2_ when deposited on different substrates, which present a unique feature of imitating growth. As shown in Fig. 1d–g, the pure PdS_2_ nanoplates present the quadrilateral shapes with the thickness of about 75 nm, which is monocrystalline by the evidence of the characteristic diffraction spot pattern. For PdS_2_@GO, PdS_2_ presents a large and thin layer structure similarly to GO, whose thickness is reduced to 12–20 nm (Fig. S6a, b), while four well-defined concentric diffraction rings appear in the SAED pattern of PdS_2_@GO corresponding to the (002), (111), (200) and (022) lattice plane (Fig. S6c, inset), respectively, demonstrating that PdS_2_ growing on GO is polycrystalline with the arbitrary crystal orientation. The irregularly shaped PdS_2_ with the thickness closed to the pure PdS_2_ nanoplates can be observed on PPy/GO in Fig. S7a, b, whose SAED pattern displays that four concentric diffraction rings are composed of many diffraction spots (Fig. S7c, inset), indicating that PdS_2_ growing on PPy/GO is polycrystalline with the limited crystal orientation.

**Fig. 3:**
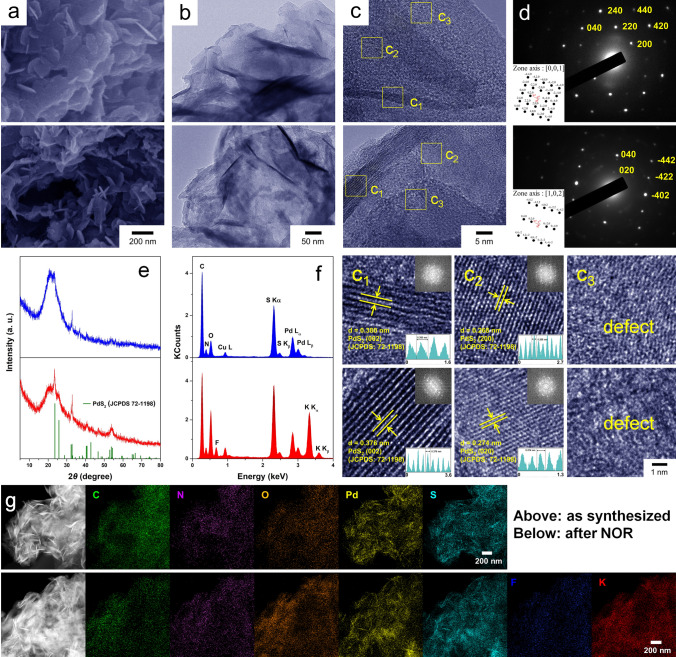
**a** SEM, **b** TEM, **c** HRTEM images (with the amplified images of the region enclosed by three yellow squares of (c_1_), (c_2_), (c_3_) and the corresponding FFT pattern), **d** SAED pattern with the corresponding simulated diffraction pattern, **e** XRD patterns, **f** EDS spectra and **g** high-angle annular dark field (HAADF) images of PdS_2_@PVEIB/PPy/GO (above: as synthesized, below: after electrolysis for 30 h in N_2_ saturated electrolyte) with the corresponding elemental mapping of C, N, O, Pd, S, F, K

Particularly, when PVEIB/PPy/GO is selected as the substrate, the well-defined thin and small PdS_2_ nanoplates with the thickness of about 25 nm are anchored on curled lamella surfaces (Fig. 3a, b) due to the induction and the confinement effect of PVEIB, which is derived from the steric hindrance constructed by imidazolium groups. Besides two lattice spacings calculated to 0.380 and 0.268 nm corresponding to the (002) and (200) lattice plane observed in the HRTEM images of PdS_2_@PVEIB/PPy/GO as synthesized with the amplified images of the region enclosed by two yellow squares of (c_1_) and (c_2_), many defects also appear in the region enclosed by the yellow squares of (c_3_), which may create more exposed active sites and further accelerate the NOR process, thus resulting in the higher NO_3_^−^ yield and FE. The two lattice spacings are consistent with results of the lattice fringe measurement by the simulated FFT in Fig. 3c1 and 3c_2_, respectively. The SAED pattern of PdS_2_@PVEIB/PPy/GO in Fig. 3d presents a typical diffraction spot pattern, well matching to the (200), (220), (240), (040), (420), and (440) lattice plane of orthorhombic PdS_2_ (JCPDS No. 72–1198), demonstrating that the thin PdS_2_ nanoplates in PdS_2_@PVEIB/PPy/GO as synthesized are monocrystalline, which are consistent with the corresponding simulated diffraction pattern (Fig. 3d). The variations in crystallinity and lamellar thickness of PdS_2_ nanoplates anchored on the different substrates may be caused by the different induction of the exposed chemical groups on the substrates, which influence the growth characteristic for PdS_2_. Comparing the XRD patterns of the pure PdS_2_ nanoplates, PdS_2_@GO, PdS_2_@PPy/GO and PdS_2_@PVEIB/PPy/GO (Figs. 1c, S6d, S7d, and 3e), a significant difference can be observed in their crystallinity. The thinner the thickness of PdS_2_ nanoplates are, the poorer their crystallinity is, resulting in more defects, which will further present better electrocatalytic NOR performance in possible. In addition, the peaks of C, N, O, Pd and S appear in Fig. 3f determined by energy-dispersive X-ray spectrometer (EDS), and the corresponding elemental mapping images in Fig. 3g present that the profiles of Pd and S element are visually differentiated from that of C, N, O element, well demonstrating the excellent inorganic/organic hierarchical 2D/2D heterostructures of PdS_2_@PVEIB/PPy/GO.

After electrolysis for 30 h in N_2_ saturated electrolyte, the morphology of PdS_2_@PVEIB/PPy/GO remains the same, while the thickness of PdS_2_ nanoplates has increased a little in Fig. 3a and b, resulting from the layer number of (002) lattice plane significantly increases after NOR, as shown in the HRTEM image (Fig. 3c), especially the amplified image of the region enclosed by the yellow squares of (c_1_). Even so, the thin PdS_2_ nanoplates in PdS_2_@PVEIB/PPy/GO after NOR are still monocrystalline, testified by the typical diffraction spot pattern also appearing in SAED pattern (Fig. 3d). Obviously seen in the XRD patterns of PdS_2_@PVEIB/PPy/GO before and after NOR, the intensity of diffraction peaks in Fig. 3e is much stronger than that in Fig. 3e, derived from the increase of the thickness of PdS_2_ nanoplates after NOR, resulting in the improved crystallinity of PdS_2_. In addition, after NOR, two additional peaks of F and K appear in Fig. 3f with the corresponding elemental mapping images in Fig. 3g, caused by the added Nafion perfluorinated resin and the adsorbed KOH. Therefore, PdS_2_@PVEIB/PPy/GO demonstrates outstanding stability in terms of morphology, crystal structure and chemical components during the NOR process, which may be one of the reasons for its exceptional stability in NOR electrocatalysis.

### Investigation of NOR Mechanism Based on PdS_2_

**Fig. 4:**
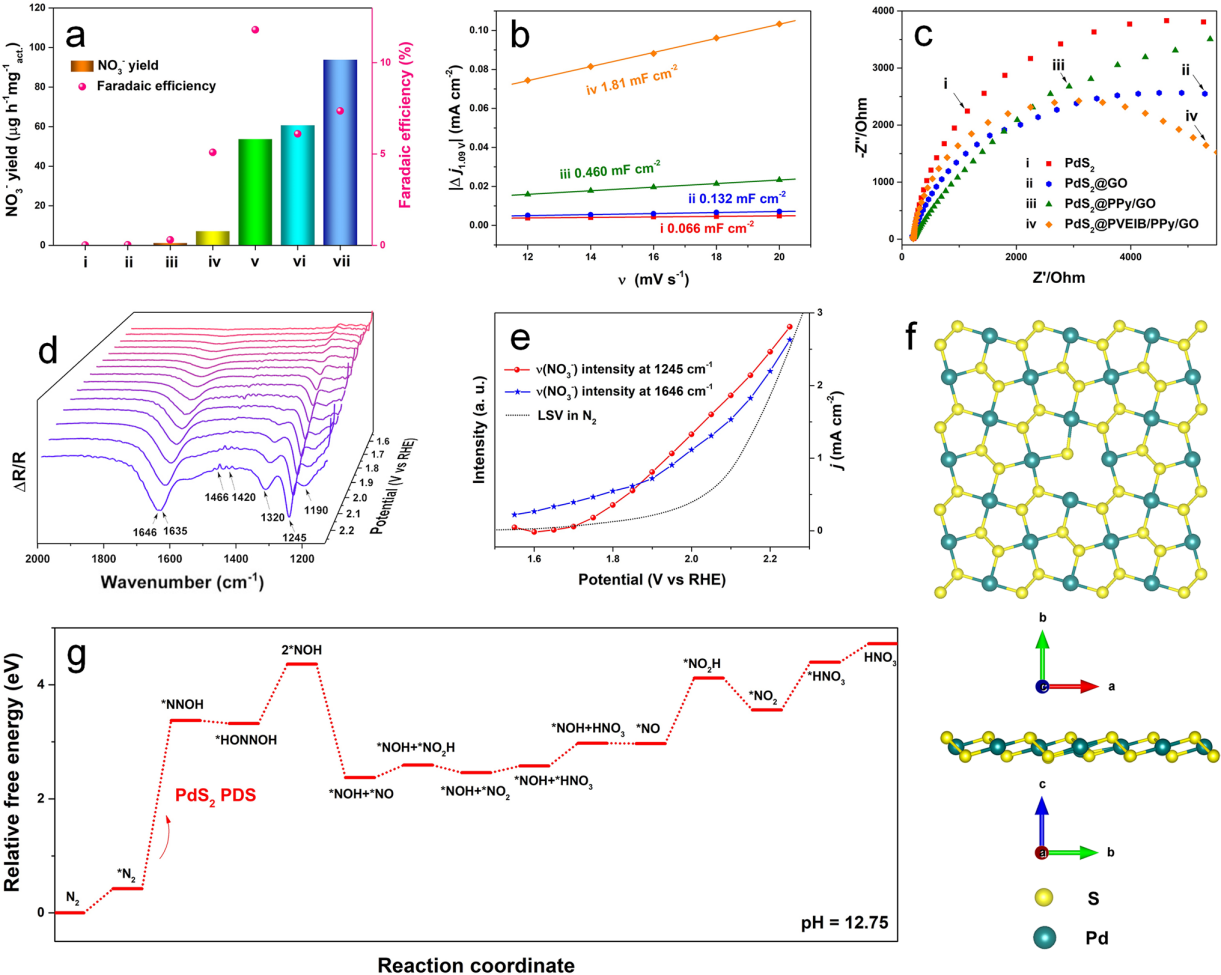
**a** NO_3_^−^ yield and FE obtained by different electrocatalysts at 2.05 V (vs. RHE) under electrolysis for 2 h: (i) GO, (ii) PPy/GO, (iii) PVEIB/PPy/GO, (iv) PdS_2_, (v) PdS_2_@GO, (vi) PdS_2_@PPy/GO and (vii) PdS_2_@PVEIB/PPy/GO; **b** C_dl_ of (i) PdS_2_, (ii) PdS_2_@GO, (iii) PdS_2_@PPy/GO and (iv) PdS_2_@PVEIB/PPy/GO modified GCE; **c** Nyquist diagrams of (i) PdS_2_, (ii) PdS_2_@GO, (iii) PdS_2_@PPy/GO and (iv) PdS_2_@PVEIB/PPy/GO modified GCE in 1 M KOH recorded at DC potential of 2.05 V (vs. RHE); **d** Electrochemical in situ ATR-SEIRAS of PdS_2_ nanoplates from 1.55 to 2.25 V (vs. RHE) in N_2_-saturated 0.1 M KOH electrolyte; **e** Potential-dependent peak intensity of NO_3_^−^ vibration (1245 and 1646 cm^−1^) derived from **d** and the corresponding LSV curve in N_2_-saturated 0.1 M KOH electrolyte; **f** Top view of the (002) surface of PdS_2_ monolayer with an S-vacancy; **g** Relative free energy diagram for NOR under alkaline conditions over the (002) surface of PdS_2_ monolayer with sulfur vacancy

In order to explore the NOR mechanism, the catalytic centers of PdS_2_@PVEIB/PPy/GO must be firstly ascertained. It is apparent that all the substrates, including GO, PPy/GO and PVEIB/PPy/GO, have almost no NOR electroactivity at 2.05 V (Fig. 4a), definitely demonstrating that PdS_2_ is real catalytic center for NOR in the all composite catalysts. Obviously, all the constructed 2D/2D heterostructures based on PdS_2_, including PdS_2_@GO, PdS_2_@PPy/GO and PdS_2_@PVEIB/PPy/GO, have exhibited the significantly improving NOR electroactivity compared to the pure PdS_2_ nanoplates. The double-layer capacitance (C_dl_) of the pure PdS_2_ nanoplates, PdS_2_@GO, PdS_2_@PPy/GO and PdS_2_@PVEIB/PPy/GO obtained from the cyclic voltammetry (CV) plots in Fig. S8 are calculated to 0.066, 0.132, 0.460 and 1.81 mF cm^−2^ in Fig. 4b. Thus, the electrochemical active surface area (ECSA) of all the electrocatalysts based on PdS_2_ can be estimated to 1.5, 3.3, 11.5 and 42.45 cm^2^/cm^2^ from their C_dl_ by Eq. S3, indicating PdS_2_@PVEIB/PPy/GO possesses the largest ECSA among them. Figure 4c presents the Nyquist diagrams of the above four electrocatalysts modified glassy carbon electrode (GCE) obtained by the electrochemical impedance spectroscopy (EIS). They all display only “one semicircle” feature at the potential of 2.05 V (vs. RHE), and the smallest semicircle domain is obviously found in the Nyquist plot of PdS_2_@PVEIB/PPy/GO in Fig. 4c-iv, implying that the charge-transfer resistance (R_ct_) can be significantly reduced by the synergistic effect of each component in PdS_2_@PVEIB/PPy/GO. It is demonstrated that a higher charge-transfer rate can be achieved by PdS_2_@PVEIB/PPy/GO, accelerating the NOR process and resulting in the better NOR electroactivity.

According to the above results, PdS_2_ nanoplates were selected for electrochemical in situ ATR-SEIRAS test to detect the reaction intermediates of NOR, availing to eliminate other interference and elucidate the mechanistic pathway underlying the electrocatalysis of N_2_ conversion to NO_3_^−^ by PdS_2_ nanoplates in alkaline conditions. The spectra of real-time ATR-SEIRAS are captured during the anodic scan of PdS_2_ nanoplates in a N_2_-saturated 0.1 M KOH solution from 1.55 to 2.25 V (vs. RHE) (Fig. 4b). Obviously, the intensity of two prominent absorption bands at 1245 and 1646 cm^−1^ corresponding to bridging nitrate exhibits a progressive increase as the potential range extends from 1.6 to 2.25 V (vs. RHE), along with the monodentate nitrate at 1635 cm^−1^ [35]. Three distinct bands associated with adsorbed N-containing oxide species are observed at approximately 1320, 1420, and 1466 cm^−1^, corresponding to bridging bidentate nitrites [36], NO_3_^−^ vibration [37] and the N = O stretching vibration of linear nitrites [35], respectively. Figure 4c presents the potential dependence of the bridging nitrate vibration at 1245 and 1646 cm^−1^ and the corresponding LSV curve (black shot dot line) under N_2_ atmosphere. Two bands exhibit a significant increase in intensity as the potential range from 1.6 to 2.25 V (vs. RHE), concurrent with the rise in oxidation current, suggesting an increased coverage of absorbed *NO_3_^−^ on PdS_2_ nanoplates. Based on the observed intermediates, the associative distal pathway for NOR is inferred through DFT calculations performed on a PdS_2_ (002) monolayer with an S-vacancy, whose structure is displayed in Fig. 4d and constructed from HRTEM measurements in Fig. 3. The calculated Gibbs free energy (Δ*G*) diagram of the NOR kinetic pathway on the S-vacancy of PdS_2_ (002) is displayed in Fig. 4e, with the atomistic structures describing the reaction pathway illustrated in Fig. S9. The step for the oxidation of *N_2_ to form *NNOH has the highest energy barrier of 2.95 eV, which is regarded as the potential-determining step (PDS) of the whole NOR process.

### Facilitated NOR Electroactivity of PdS_2_ by In Situ Generated Sulfate

**Fig. 5:**
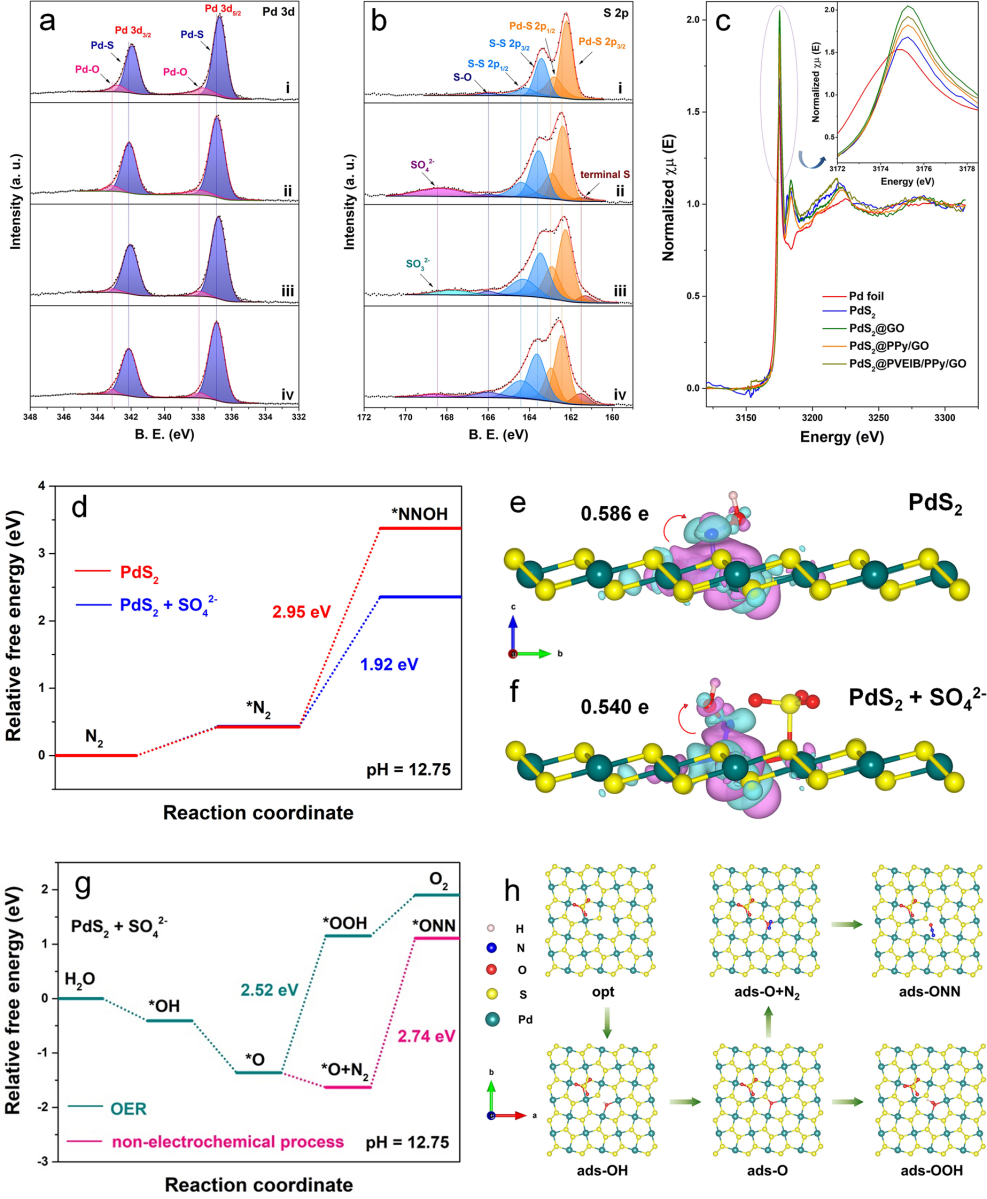
**a** Pd 3*d* and **b** S 2*p* spectra by XPS of (i) PdS_2_ nanoplates, (ii) PdS_2_@GO, (iii) PdS_2_@PPy/GO and (iv) PdS_2_@PVEIB/PPy/GO; **c** Normalized Pd L_3_-edge HERFD-XANES spectra for various electrocatalysts based on PdS_2_; **d** Relative free energy diagram of N_2_ electrooxidation process on PdS_2_ and PdS_2_ + SO_4_^2−^; charge density difference of *NNOH adsorbed on **e** PdS_2_ and **f** PdS_2_ + SO_4_^2−^, where the isosurface value is set to be 0.002 e Å^−3^ and the magenta and cyan isosurfaces stand for the electron depletion and accumulation, respectively; **g** Relative free energy diagram OER process and **h** the corresponding atomistic structure scheme for describing the reaction pathway of OER on PdS_2_ + SO_4_^2−^, as well as the non-electrochemical process of NOR

Previous theoretical calculations have indicated that monolayer PdS_2_ behaves as a typical semiconductor, while bilayer PdS_2_ can exhibit the semimetallic property [38]. It is inferred that the transition of PdS_2_ from a semiconductor to a semimetal will occur with an increase in the number of layers [39]; in other words, the thicker the PdS_2_ nanoplates are, the enhancer the semimetallic property they have. Therefore, this unique imitating growth feature not only has an effect on the thickness and shape of PdS_2_, but also significantly affects its electronic state, which can be confirmed by XPS, and finally resulting in the differences to its NOR performance. The Pd 3*d* and S 2*p* XPS spectra of the pure PdS_2_ nanoplates, PdS_2_@GO, PdS_2_@PPy/GO and PdS_2_@PVEIB/PPy/GO are displayed in Fig. 5a, b. Because the PdS_2_ nanoplates with thinner thickness and more defects anchored on GO and PVEIB/PPy/GO are easy to be oxidized, the higher bonding energy of Pd 3*d*_5/2_ and Pd 3*d*_3/2_ orbitals of Pd-S bonding appears at 336.9 and 342.1 eV [40] (Fig. 5a-ii and iv), respectively, while the pure PdS_2_ nanoplates and PdS_2_ nanoplates anchored on PPy/GO present the enhanced semimetallic property due to the increased thickness, resulting in the lower bonding energy of Pd 3*d*_5/2_ and Pd 3*d*_3/2_ orbitals of Pd-S bonding in Fig. 5a-i and iii.

Due to the unique imitating growth feature, the oxidation degree of PdS_2_ nanoplates is affected by thickness and defects during the preparation process or exposed in air, and these differences are visible in S 2*p* spectra in Fig. 5b. The peak at 168.6 eV corresponding to SO_4_^2−^ [41] can be detected in the S 2*p* spectra of PdS_2_@GO and PdS_2_@PVEIB/PPy/GO (Fig. 5b-ii and iv), caused by the in situ oxidation of the defects on the thinner PdS_2_ nanoplates during the preparation process or exposed in air. Especially, PdS_2_@GO presents the obviously stronger peak of SO_4_^2−^, compared to PdS_2_@PVEIB/PPy/GO, indicating the higher oxidation degree of PdS_2_ nanoplates on GO, which may be caused by the thinner the PdS_2_ nanoplates are, the more defects they have, and the easier they are to be oxidized. No peak related to SO_4_^2−^ can be fitted in the S 2*p* spectra of the pure PdS_2_ nanoplates and PdS_2_@PPy/GO (Fig. 5b-i and iii), due to the enhanced thickness of PdS_2_ nanoplates with better crystallinity and less defects. The above difference of the oxidation degree of PdS_2_ nanoplates anchored on different substrates can be also verified by their Pd L_3_-edge high energy resolution fluorescence detected X-ray absorption near-edge (HERFD-XANES) spectra (Fig. 5c). It can be clearly observed that the position of the main, intense white line shifts to higher energy from Pd foil to PdS_2_, while the white line intensity follows the order of PdS_2_ < PdS_2_/PPy/GO < PdS_2_@PVEIB/PPy/GO < PdS_2_@GO, further demonstrating that the oxidation degree of PdS_2_ nanoplates anchored on GO and PVEIB/PPy/GO is higher than that anchored on PPy/GO, and the lowest is the pure PdS_2_ nanoplates.

Based on the above results and the differences in the NO_3_^−^ yield and FE obtained by the four electrocatalysts in Figs. 1h and 2c, it can be inferred that the existence of in situ generated SO_4_^2−^ would remarkably promote the NOR electroactivity of PdS_2_. In order to further verify this conjecture, a PdS_2_ + SO_4_^2−^ structure model has been optimized by the in situ generated SO_4_^2−^ on a (002) surface of PdS_2_ monolayer with an S-vacancy, as shown in Fig. S10. The calculated Gibbs free energy (Δ*G*) diagram of N_2_ electrooxidation process on PdS_2_ and PdS_2_ + SO_4_^2−^ is presented in Fig. 5d. Obviously, the energy barrier for the oxidation of *N_2_ to form *NNOH on PdS_2_ + SO_4_^2−^ reduces to 1.92 eV by DFT calculations, indicating that the in situ generated SO_4_^2−^ can effectively reduce the activation energy of the PDS, thus accelerating NOR [16]. Subsequently, the charge density difference of NNOH adsorbed on PdS_2_ and PdS_2_ + SO_4_^2−^ is examined to gain a deeper insight into the factors, contributing to the improved NOR activity of the catalysts. As shown in Fig. 5e and f, the NNOH adsorbed on the Pd site of PdS_2_ + SO_4_^2−^ (0.540 e) demonstrates less charge exchange and transfer between NNOH and the Pd atoms than that of NNOH adsorbed on the Pd site of PdS_2_ (0.586 e), indicating that the presence of in situ generated SO_4_^2−^ can change the amount of electron transfer from the surface to the reaction intermediate, thus reducing the energy barrier and accelerating NOR.

As is well known, electrocatalytic NOR involves activation and cleavage of N≡N with high bonding energy (941 kJ mol^−1^) [42], which is very difficult to perform kinetically and inevitably hindered by the competition from OER in aqueous solutions containing electrolytes [43]. For PdS_2_@PVEIB/PPy/GO, the existence of SO_4_^2−^ can effectively reduce the energy barrier of the PDS (the oxidation of *N_2_ to form *NNOH) at the defects of PdS_2_ (Fig. 4c), but OER is also likely to occur at this active site, calculated by DFT in Fig. 5g. Though the appropriate amount of sulfate ions (originated from chemical modification in the preparation process of the electrocatalysts or physical absorption from the electrolyte containing SO_4_^2−^) existing on the surface of electrocatalysts, can be also beneficial to stabilize the intermediate of *OOH, and thus improve the OER performances [44, 45], the PDS of OER at PdS_2_ + SO_4_^2−^ is the step for the combination of *O and OH^−^ to form *OOH with the energy barrier of 2.52 eV, which is higher than the PDS of NOR on the same active site (1.92 eV), indicating that this active site is more prone to NOR. In addition, as claimed in previous works, the non-electrochemical step of NOR is closely related to the presence of weakly adsorbed oxygen species (*O) generated from the competitive OER on the surface of electrocatalysts [46]. *O can react with N_2_ in a non-electrochemical process (*O + N_2_ → *ONN) [47]. For the constructed PdS_2_ + SO_4_^2−^, the energy barrier for the formation of *ONN is calculated to 2.74 eV, which is higher than the PDS of OER (2.52 eV) at this active site, suggesting that the occurrence of NOR is dominated by the electrochemical oxidation process of N_2_. The corresponding atomistic structure scheme for describing the reaction pathway of OER on PdS_2_ + SO_4_^2−^ is presented in Fig. 5h, as well as the non-electrochemical process of NOR.

**Fig. 6:**
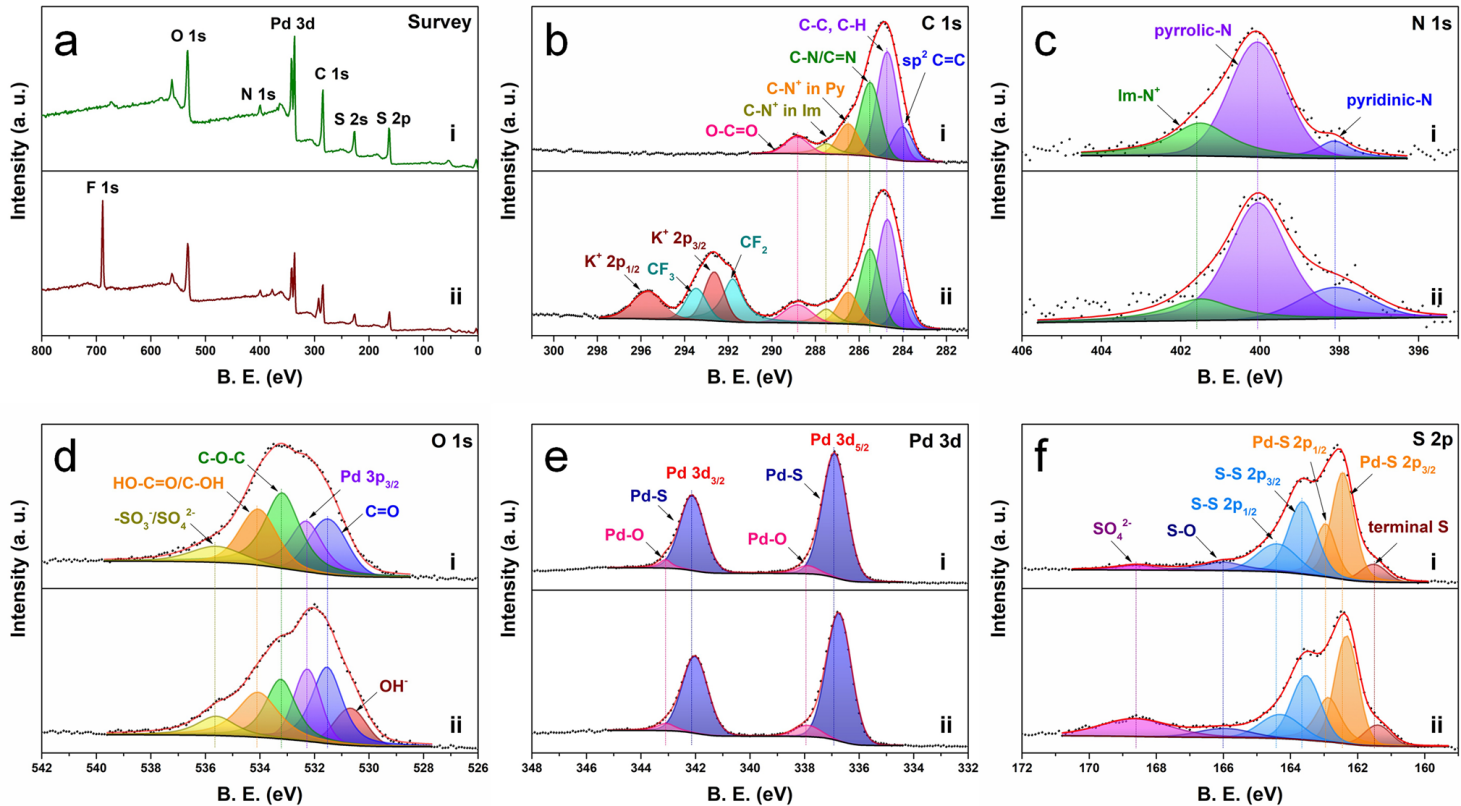
XPS spectra of PdS_2_@PVEIB/PPy/GO (i) before and (ii) after electrolysis for 30 h in N_2_ saturated electrolyte: **a** survey, **b** C 1*s*, **c** N 1*s*, **d** O 1*s*, **e** Pd 3d and **f** S 2*p*

XPS analysis can provide additional confirmation of the chemical states of PdS_2_@PVEIB/PPy/GO before and after electrolysis for 30 h in N_2_ saturated electrolyte. For PdS_2_@PVEIB/PPy/GO as synthesized, C 1*s*, N 1*s*, O 1*s*, Pd 3*d*, S 2*s,* and S 2*p* peaks are detected in its survey spectrum (Fig. 6a-i). The fitted C 1*s*, N 1*s,* and O 1*s* peaks in Fig. 6b-i, 6c-i and 6d-i can well verify the existence of GO, PPy and PVEIB, listed in Table S3 in detail. Especially, a peak fitted at 401.5 eV ascribed to N^+^ in the imidazolium ring appeared in the N 1*s* XPS spectra in Fig. 6c-i, which can well testify that PVIEB has been linked on the surface of PPy/GO. The F 1*s* peak and two peaks at 291.8, 293.5 eV belonging to CF_2_, CF_3_ groups [48, 49] are detected in the survey and C 1*s* spectra of PdS_2_@PVEIB/PPy/GO after NOR (Figs. 6a-ii and b-ii), respectively, caused by the added Nafion perfluorinated resin, while two peaks attributed to K^+^ 2*p*_3/2_ (292.6 eV) [50] and K^+^ 2*p*_1/2_ (295.7 eV) [51], as well as an additional peak related to OH^−^ (530.7 eV) [52] appear in C 1*s* and O 1*s* spectra of PdS_2_@PVEIB/PPy/GO after NOR (Fig. 6b-ii and Fig. 6d-ii), respectively, resulting from the adsorbed KOH. It is noteworthy that both peaks associated to the Pd 3*d*_5/2_ and Pd 3*d*_3/2_ orbitals of Pd-S bonding exhibit a negative shift of approximately 0.1 eV in the Pd 3*d* spectrum of PdS_2_@PVEIB/PPy/GO after NOR (Fig. 6e-ii). It may be caused by the increase in the number of PdS_2_ layers, which leads to the enhanced semimetallic property of PdS_2_, thus allowing the electrocatalyst to sustain a high NOR electroactivity. In the S 2*p* region of PdS_2_@PVEIB/PPy/GO as synthesized (Fig. 6f-i), the peaks corresponding to terminal S (161.5 eV) [53], Pd-S (162.4, 163.0 eV) [54] and bridging S (163.6, 164.4 eV) [55, 56] are well consistent with the bonding modes of the S element in PdS_2_, respectively, which also negatively shift by about 0.1 eV in Fig. 6f-ii, similarly suggesting the enhanced semimetallic property of PdS_2_. In addition, the peak intensity of SO_4_^2−^ located at 168.6 eV [41] is significantly enhanced in Fig. 6f-ii, derived from the in situ oxidation of S in PdS_2_ at the high NOR potential, which can help to remain the lower activation energy of the PDS and extend the high NOR electroactivity of PdS_2,_ ultimately resulting in the excellent NOR stability of PdS_2_@PVEIB/PPy/GO.

## Conclusions

In summary, the catalytic activity and stability of PdS_2_ nanoplates toward NOR were significantly enhanced when anchored on different substrates to form 2D/2D heterostructures due to its unique imitating growth feature. The obtained PdS_2_@PVEIB/PPy/GO exhibited the excellent NOR electroactivity with the highest NO_3_^−^ yield of 93.91 μg h^−1^ mg^−1^_act._ and the maximum FE of 7.36% at 2.05 V (vs. RHE), as well as the outstanding stability and selectivity. The improved NOR performance could be contributed by the synergistic effect of each component, including PPy for facilitating electron transport, GO for providing large surface area, PVEIB for inducing defects in PdS_2_ nanoplates and the thin PdS_2_ nanoplates with the excellent activation capacity to N_2_ for acting as catalytic centers. The in situ generation of SO_4_^2−^ caused by the oxidation of thin and small PdS_2_ nanoplates with defects during the preparation process or exposed in air, as well as at high NOR potential, was found to reduce the activation energy of the reaction, accelerating NOR. This research would provide valuable insights for the development of novel electrocatalysts based on NTMDs for the applications in the electrosynthesis of nitrate.

## Supplementary Information

Below is the link to the electronic supplementary material.

Supplementary file1 (DOC 25099 KB)
