# Supplementary material for: In Situ Generated Sulfate-Facilitated Efficient Nitrate Electrosynthesis on 2D PdS2 with Unique Imitating Growth Feature
Source: Nanomicro Lett. 2025 Jun 12;17:289. doi: 10.1007/s40820-025-01803-3 (PMC12158905; doi:10.1007/s40820-025-01803-3)
Supplement: Supplementary file 1 — Supplementary file1 (DOC 25099 KB) [file 40820_2025_1803_MOESM1_ESM.doc]

# Supporting Information for

# In Situ Generated Sulfate-Facilitated Efficient Nitrate Electrosynthesis on 2D PdS_2_ with Unique Imitating Growth Feature

# Rui Zhang ^1, 2^†, Hui Mao^3 *^†, Ziyi Wang^3^, Shengke Ma^3^, Shuyao Wu^3^, Qiong Wu^3^, Daliang Liu^3^, Hui Li^2, 4^, Yang Fu ^2, 4^, Xiaoning Li^2, 4^, and Tianyi Ma^2, 4 *^

# ^1^ Shenyang Key Laboratory of Medical Molecular Theranostic Probes in School of Pharmacy, School of Pharmacy, Shenyang Medical College, Shenyang 110034, P. R. China

# ^2^ Centre for Atomaterials and Nanomanufacturing (CAN), School of Science, RMIT University, Melbourne VIC 3000, Australia

# ^3^ Institute of Clean Energy Chemistry, Liaoning Key Lab for Green Synthesis and Preparative Chemistry of Advanced Materials, College of Chemistry, Liaoning University, Shenyang 110036, P. R. China

# ^4^ ARC Industrial Transformation Research Hub for Intelligent Energy Efficiency in Future Protected Cropping (E2Crop), Melbourne, VIC 3000, Australia

† Rui Zhang and Hui Mao Contributed equally to this work.

* Corresponding authors. E-mail: [huimao@lnu.edu.cn](mailto:huimao@lnu.edu.cn) (Hui Mao); [tianyi.ma@rmit.edu.au](mailto:tianyi.ma@rmit.edu.au) (Tianyi Ma)

**S1 Experimental Section**

**S1.1 Materials**

Pyrrole (Py) (≥98.0%), bromoethane (≥98.0%), allyl chloride (≥98.0%) and high-purity graphite powder (≥99.85%) are chemical grade, purchased from Sinopharm Chemical Reagent Co. Ltd., while KOH was guaranteed reagent (GR). Nafion perfluorinated resin solution (Nafion, 5 wt% in lower aliphatic alcohols and water) was purchased from Aldrich. N-vinyl imidazole was purchased from Yancheng Medical Chemical Factory (China), which was distilled under vacuum before use. All the other reagents were analytical grade and used without further purification, such as KMnO_4_ (Tianjin Baishi Chemical Co. Ltd, ≥99.5%), FeCl_3_·6H_2_O (≥99.0%) and thioacetamide (TAA, ≥99.0%) (Sinopharm Chemical Reagent Co. Ltd.), K_2_PbCl_6_ (Pd = 26.2%) and 2,2-azobisisobutyronitrile (AIBN) (≥98.0%) (Aladdin reagent (Shanghai) Co., Ltd.), ethanol (≥99.7%) and NaHCO_3_ (≥99.5%) (Tianjin Damao Chemical Factory), dimethyl formamide (DMF) (≥99.5%) and NaNO_3_ (≥99.0%) (Tianjin Beilian Fine Chemicals Development Co., Ltd.), H_2_O_2_ (Shenyang Xinhua Reagent Factory, ≥30.0%). Ultra-pure water was purchased from Wahaha Group Co., Ltd. (Hangzhou, China).

**S1.2 Preparation of GO, PPy/GO and PVEIB/PPy/GO Nanosheets**

GO were obtained and purified by Hummers method [S1]. PPy/GO nanosheets were prepared by a typical ultra-sound way [S2], where the mass ratio of GO and Py was 1:1. PVEIB/PPy/GO nanosheets were prepared by our previous method [3]. Firstly, vinyl radicals were fixed on the surface of PPy/GO as our previous report [S4], and then VEIB (synthesized according to the previous literature [S5]) were polymerized on their surface, obtaining PVEIB/PPy/GO nanosheets. The black products were collected by centrifugation, washed several times with water and ethanol, finally dried under vacuum at 45 °C for 24 h.

**S1.3 Preparation of** **GCE Modified by the Electrocatalysts Based on PdS_2_**

The glassy carbon electrode (GCE) modified by the electrocatalysts based on PdS_2_ was prepared by the same method. Taking PdS_2_ nanoplates as an example, the PdS_2_ nanoplates suspension was prepared by adding 1.5 mg of PdS_2_ nanoplates into 460 μL ethanol mixed with 40 μL Nafion perfluorinated resin solution, and ultrasound dispersed evenly for 5 min. Then, 5 μL of suspension were dropped on the surface of a GCE (φ = 3 mm) to form a layer and drying in the environment. The modified GCE was used as the working electrode for linear sweep voltammetry (LSV) and cyclic voltammetry (CV) measurement.

**S1.4 Characterizations and Apparatus**

The morphology and size of all samples, such as PdS_2_, PdS_2_@GO, PdS_2_@PPy/GO and PdS_2_@PVEIB/PPy/GO, were assessed by using a Hitachi SU-8010 field emission scanning electron microscope (FESEM) working at 10 kV. Transmission electron microscopy (TEM) image and high-resolution TEM (HRTEM) image of PdS_2_@PVEIB/PPy/GO were determined by FEI Talos F200X G2 transmission electron microscope equipped with a CCD camera at an acceleration voltage of 200 kV. The element mapping analysis of PdS_2_@PVEIB/PPy/GO was also determined by FEI Talos F200X G2 transmission electron microscope with an energy-dispersive X-ray spectrometer (Super-X EDS). A JEM-2100 electron microscope (JEOL, Japan) equipped with a CCD camera at an acceleration voltage of 200 kV was used to obtain the TEM and HRTEM images of PdS_2_, PdS_2_@GO and PdS_2_@PPy/GO, as well as the selected area electron diffraction (SAED) patterns of all samples. The electron diffraction (ED) patterns of PdS_2_ and PdS_2_@PVEIB/PPy/GO were also simulated using the CaRIne Crystallography 3.1 and compared with those obtained by the interpretation. X-Ray Diffraction (XRD) patterns were obtained with a D8 AXS diffractometer (Bruker, GER) using Cu Kα radiation. Analysis of the X-ray photoelectron spectra (XPS) was performed on a Kratos Axis Ultra DLD electron spectrometer using a monochromatic Al Kα source operated at 150 W. The weight content of Pd element in all of samples were determined by an inductively coupled plasma-optical emission spectrometry (ICP-OES, Agilent 5110, Agilent, USA) after concentrated acids (HNO_3_:H_2_O_2_=7:1, v/v) digestion. High energy resolution fluorescence detected X-ray absorption near-edge (HERFD-XANES) measurements were carried out using the Medium Energy X-ray Absorption Spectroscopy at beamline MEX-2 (monochromatic 1.7 – 3.5 keV). This branch line will offer low energy monochromatic X-rays from either InSb (111) or Si (111) double crystal monochromators at a fixed position and with a maximum beam size of 2 x 5 mm^2^. Slits can be used to reduce the beam profile on the sample. Fluorescence from the sample is diffracted by a cylindrically bent, Si (111) Johann analyzer in a dispersive Rowland refocusing (DRR) geometry. The variable temperature sample environment can be from 10 to 300 K under helium gas atmosphere and/or vacuum.

**S1.5 Nitrate Detection**

The produced NO_3_^-^ was determined by an ion chromatography (IC) (Dionex ICS-900 Starter Line IC System, Thermo Scientific, USA) with an anion column (Dionex IonPac AS19, Thermo Scientific, USA). The standard curve is constructed by measuring a series of specific conductance for the reference solutions with different KNO_3_ (standard substance, GSB 04-1772-2004) concentrations (0.0 μg mL^-1^, 0.1 μg mL^-1^, 0.2 μg mL^-1^, 0.3 μg mL^-1^, 0.5 μg mL^-1^, 1.0 μg mL^-1^, 2.0 μg mL^-1^, 3.0 μg mL^-1^) in KOH. The fitting curve (y = 0.3062x + 0.00233，R^2^ = 0.9995) shows good linear relation of peak area with KNO_3_ concentration, where the peak area of KOH is deducted.

**S1.6 Nitrite Detection**

The produced NO_2_^-^ was also determined by an Ion chromatography (IC) (Dionex ICS-900 Starter Line IC System, Thermo Scientific, USA) with an anion column (Dionex IonPac AS19, Thermo Scientific, USA). The standard curve is constructed by measuring a series of specific conductance for the reference solutions with different NaNO_2_ (standard substance, GSB 04-2839-2011) concentrations (0.1 μg mL^-1^, 0.2 μg mL^-1^, 0.3 μg mL^-1^, 0.5 μg mL^-1^, 1.0 μg mL^-1^, 2.0 μg mL^-1^, 3.0 μg mL^-1^) in water. The fitting curve (y = 0.3429x - 0.003，R^2^ = 0.9999) shows good linear relation of peak area with NaNO_2_ concentration.

**S1.7 Computational Formula**

The NO_3_^-^ yield rate was calculated using the following equation:

NO_3_^-^ yield rate = [NO_3_^-^]×V/(m×t) （S1）

where [NO_3_^-^] is the concentration of the produced NO_3_^-^, V is the volume of the electrolyte, t is the chronoamperometry test time and m is m_cat_. (the mass of the catalyst) or m_act_. (the mass of the active constituent in catalyst) (for PdS_2_@PVEIB/PPy/GO, m_cat_. = m_PdS2_ + m_PVEIB/PPy/GO_, and m_act_. = m_PdS2_).

The Faradaic efficiency (FE) was obtained by dividing the total charge used for the electrodes by the quantity of electric charge for nitrate production, assuming that five electrons were consumed to produce one NO_3_^-^ ion. FE was calculated according to the following equation:

FE = 5×F×[NO_3_^-^]×V/(62×Q) (S2)

where F is the Faraday constant (96485 C mol^-1^), [NO_3_^-^] is the concentration of the produced NO_3_^-^, Q is the quantity of applied electricity and V is the volume of the electrolyte.

**S1.8 Double Layer Capacitance (C_dl_) Measurements:** To measure the electrochemical capacitance, the potential was swept between 0.95 to 1.25 V vs. RHE at different scanning rates (12, 14, 16, 18, 20 mV s^-1^) with an assumption of double layer charging in the potential range. The capacitive currents at 1.09 V vs. RHE were measured and plotted as a function of scanning rate. The linear slope of |Δj| = |j_a_ − j_c_| against scanning rates was twice the C_dl_.

**S1.9 Determination of Electrochemically Active Surface Area (ECSA):** Assume that the specific capacitance (C_s_) for a flat surface was about 40 μF cm^−2^ for 1 cm^2^ of real surface area, the ECSA was calculated as follows:

ECSA = C_dl_/C_s_ (S3)

**S1.10 Electrochemical in situ Attenuated Total Reflection Surface-enhanced Infrared Absorption Spectroscopy (ATR-SEIRAS)**

The surface enhanced infrared absorption spectroscopy (SEIRAS) with the attenuated total reflection (ATR) configuration was employed. A Thermo Nicolet 8700 spectrometer equipped with MCT detector cooled by liquid nitrogen was employed for the electrochemical ATR-SEIRAS measurements. Chemical deposition of Au thin film (~60 nm) on the Si prism was prepared according to “two-step wet process”. Before chemical deposition of Au, the Si prism surface for IR reflection was polished with Diamond suspension and cleaned in water with sonication. Then the prism was soaked in a piranha solution (7:3 volumetric ratio of 98% H_2_SO_4_ and 30% H_2_O_2_) for 2 h. 1.5 mg of PdS_2_ and 40 μL of 6 % polytetrafluoroethylene solution were sonicated in 460 μL of ethanol by ultrasound treatment to generate a homogeneous ink. 30 μL of the ink was deposited and dried on the Au-film working electrode, then the ink-coated prism was assembled into a homemade spectroelectrochemical cell as the working electrode, Hg/HgO was used as reference, which was introduced near the working electrode via a Luggin capillary, a Pt mesh (1 cm × 1 cm) was serving as the counter electrode. All spectra were shown in the following equation:

$$\frac{\Delta R}{R}=\frac{E_{\text{s}}-E_{R}}{E_{R}}$$

(S4)

with Es and E_R_ representing the sample and reference spectra, respectively. The spectral resolution was 4 cm^-1^ for all the measurements if not otherwise mentioned.

**S1.11 Computational Method**

We have employed the first-principles tool, Vienna Ab initio Simulation Package (VASP) [S6, S7], to perform all density functional theory (DFT) calculations within the generalized gradient approximation (GGA) using the Perdew-Burke-Ernzerhof (PBE) [S8] formulation. We have chosen the projected augmented wave (PAW) potentials [S9, S10] to describe the ionic cores and take valence electrons into account using a plane wave basis set with a kinetic energy cutoff of 500 eV. Partial occupancies of the Kohn−Sham orbitals were allowed using the Gaussian smearing method and a width of 0.05 eV. For the optimization of both geometry and lattice size, the Brillouin zone integration was performed with a 1 × 1 × 1 Gamma centered sampling [S11]. The self-consistent calculations applied a convergence energy threshold of 10^-5^ eV. The equilibrium geometries and lattice constants were optimized with maximum stress on each atom within 0.02 eV Å^-1^. The 17 Å vacuum layer was normally added to the surface to eliminate the artificial interactions between periodic images. The weak interaction was described by DFT+D3 method using empirical correction in Grimme’s scheme [S12, S13]. Spin polarization method was adopted to describe the magnetic system. The Gibbs free energy for each elementary step was calculated as: G = E_elec_ + E_ZPE_ – TS, in which E_elec_ is the electronic energy at 0 K calculated by DFT, E_ZPE_ is the zero-point energy term, and T is the absolute temperature (here 298.15 K).

**S2 Supplementary Figures**


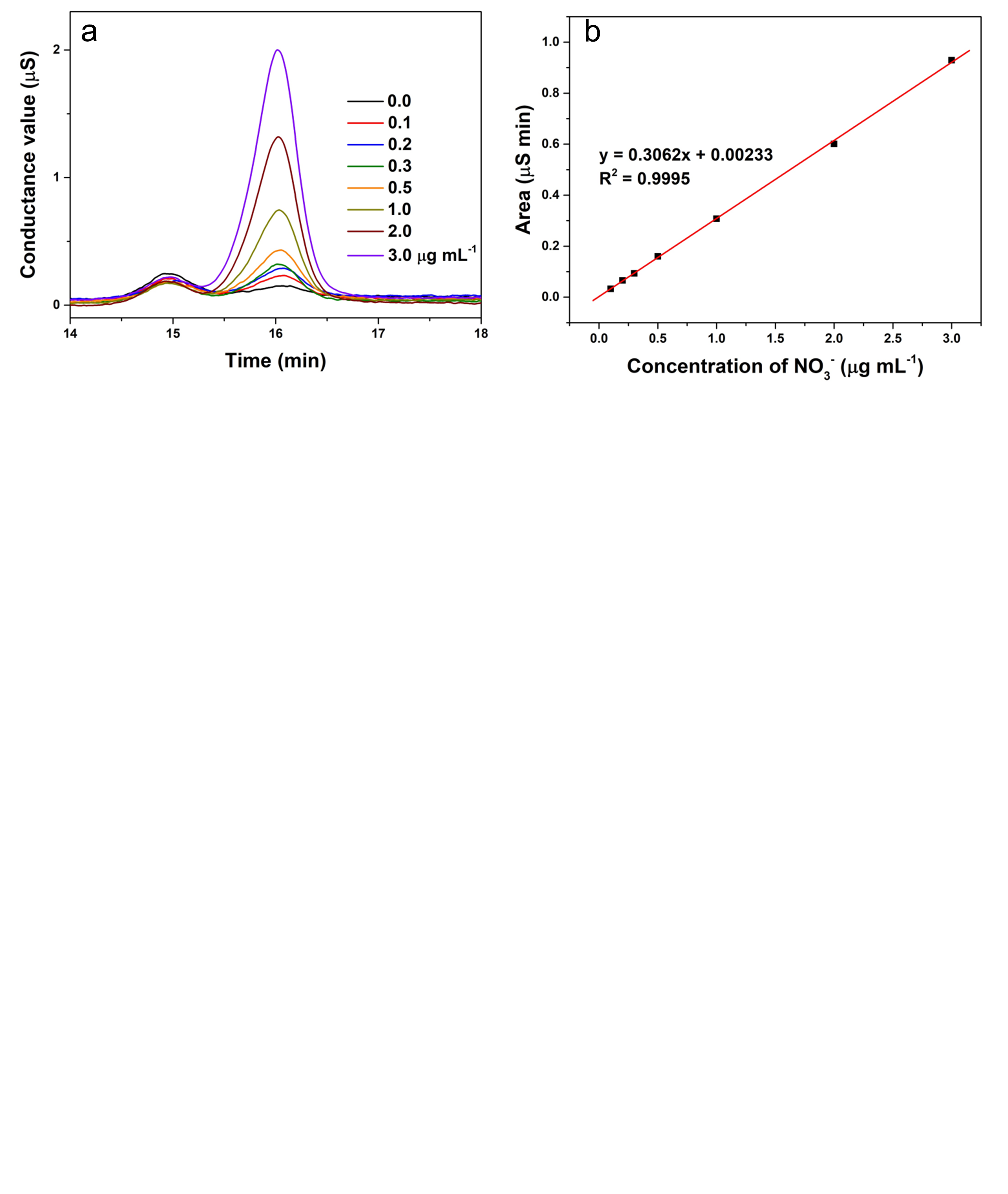


**Fig. S1** (a) Ion chromatogram spectra of the standard NO_3_^-^ concentrations; (b) calibration curve used for calculation of NO_3_^-^ concentrations


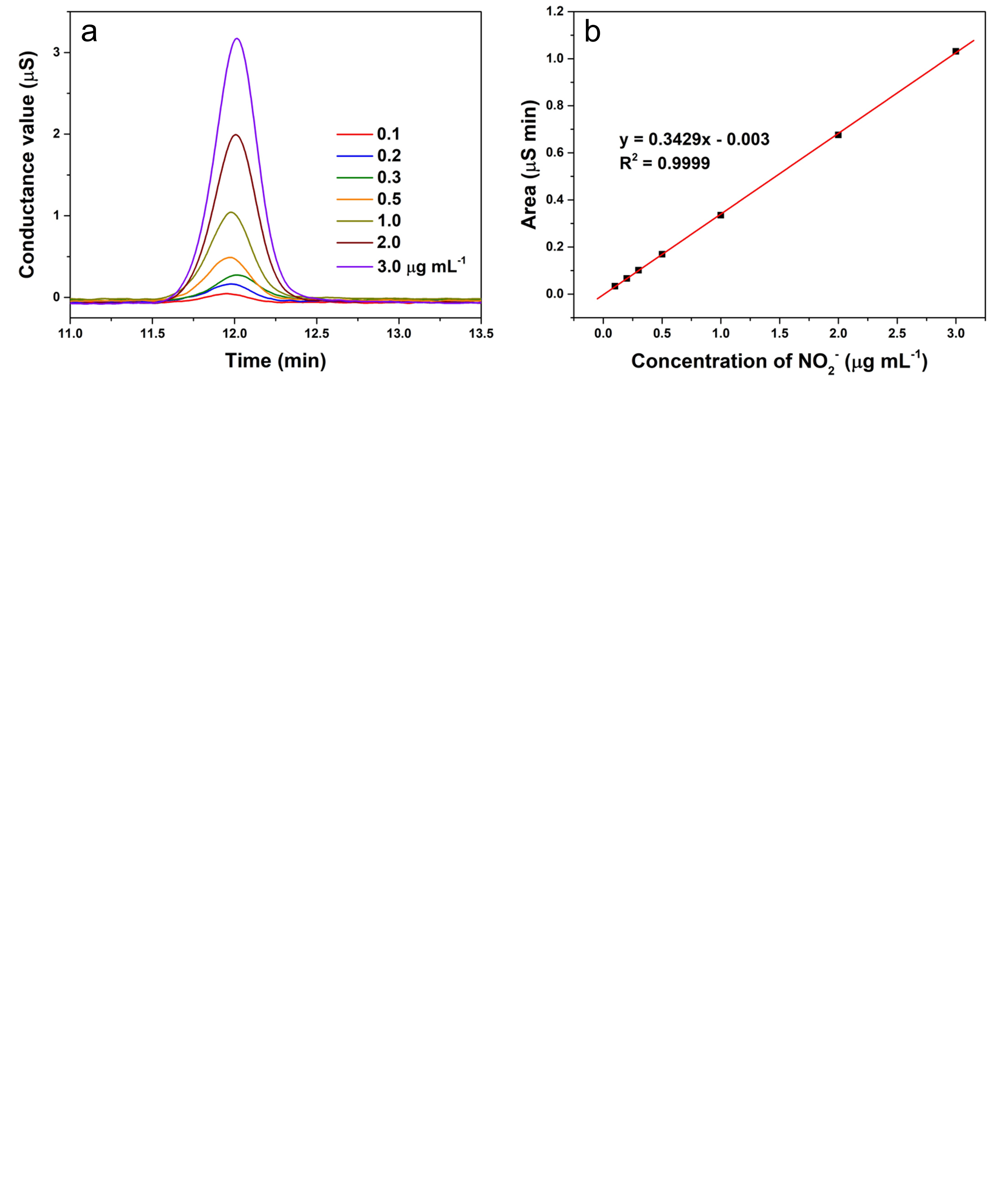


**Fig. S2** (**a**) Ion chromatogram spectra of the standard NO_2_^-^ concentrations; (**b**) calibration curve used for calculation of NO_2_^-^ concentrations


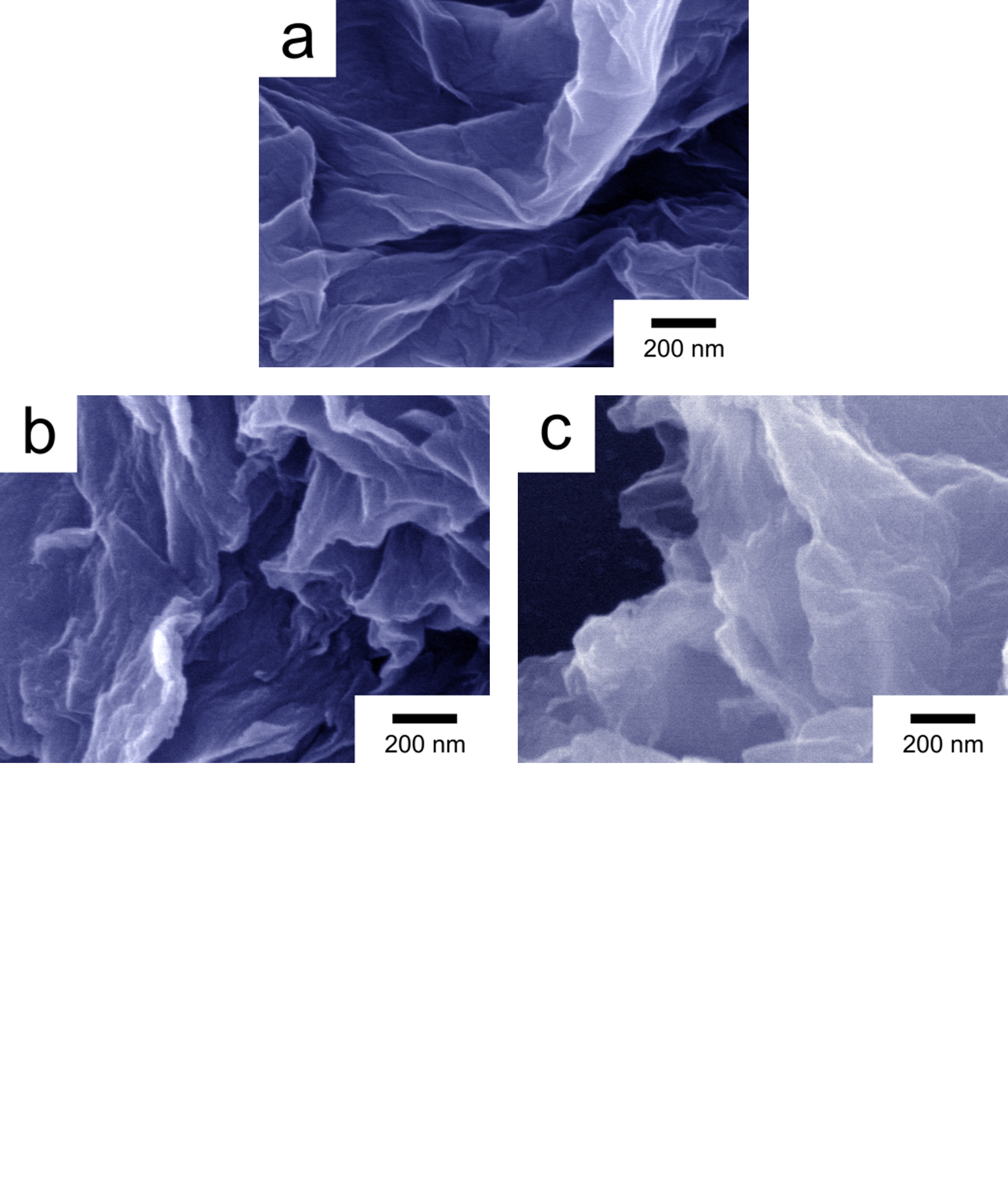


**Fig. S3** SEM images of (a) GO; (b) PPy/GO and (c) PVEIB/PPy/GO


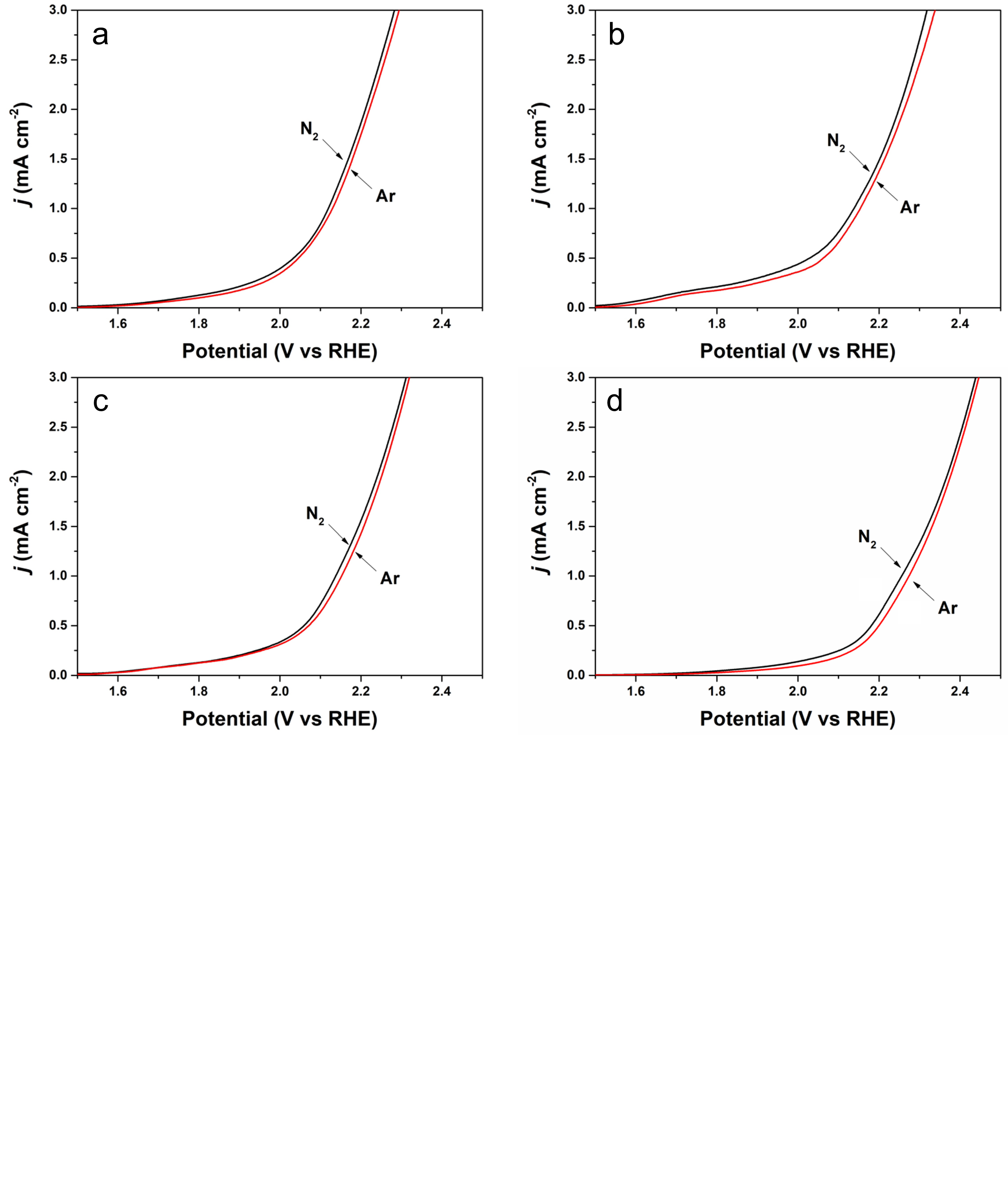


**Fig. S4** Linear sweep voltammograms of (**a**) PdS_2_, (**b**) PdS_2_@GO, (**c**) PdS_2_@PPy/GO and (**d**) PdS_2_@PVEIB/PPy/GO under saturated Ar and N_2_ in 0.1 M KOH


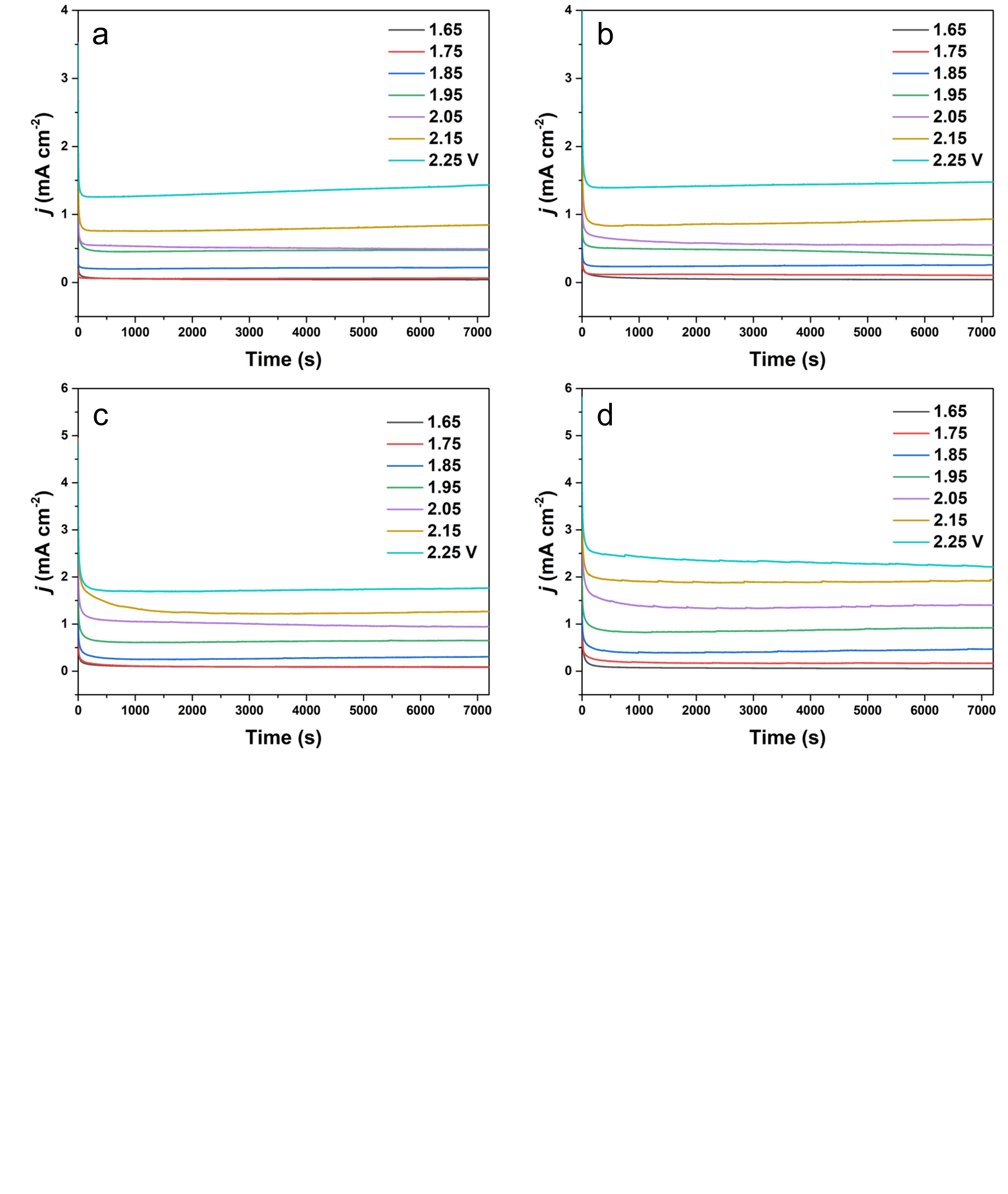


**Fig. S5** The chronoamperometry curves of (**a**) PdS_2_, (**b**) PdS_2_@GO, (**c**) PdS_2_@PPy/GO and (**d**) PdS_2_@PVEIB/PPy/GO obtained at different potentials (vs. RHE)


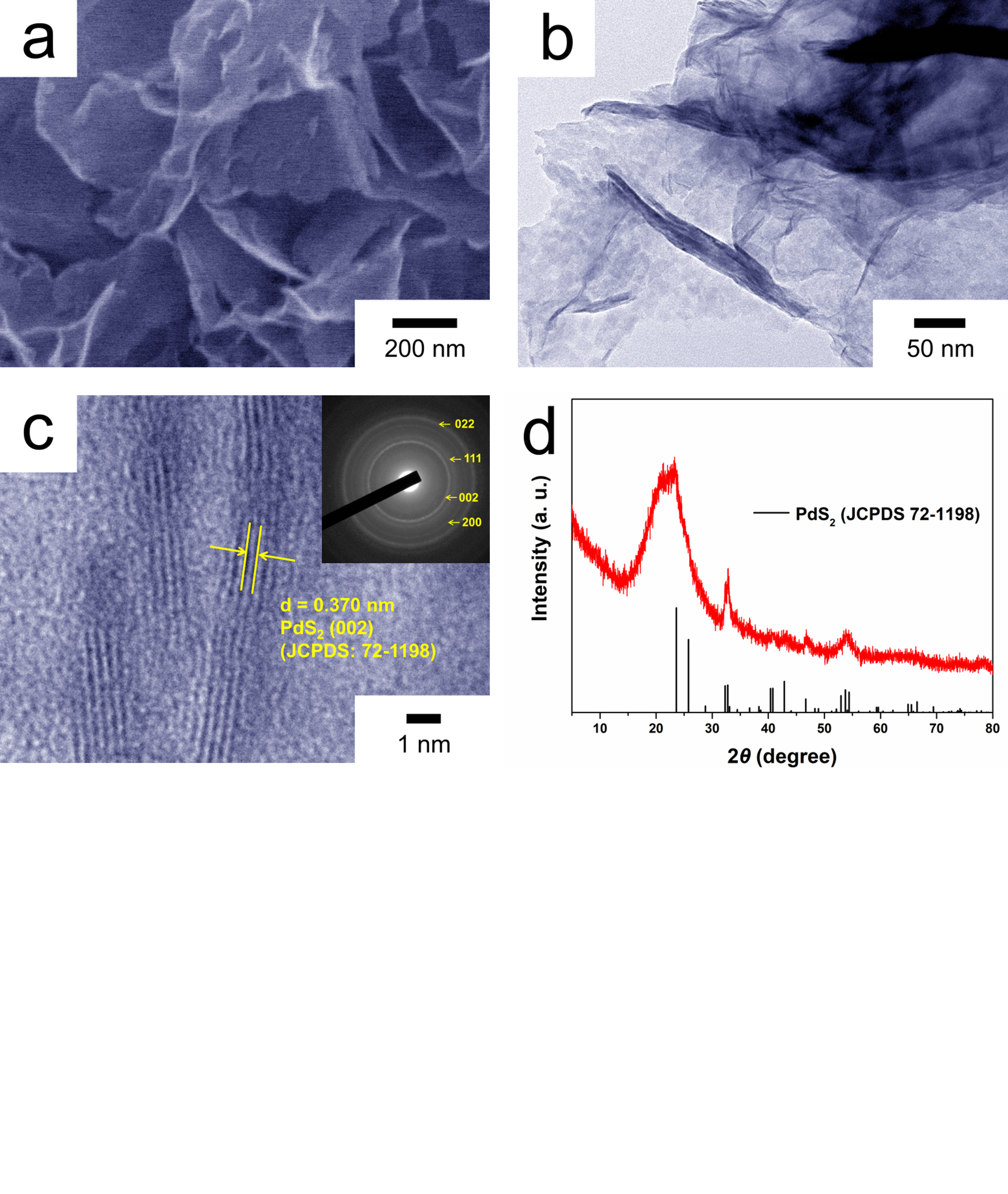


**Fig. S6** (**a**) SEM; (**b**) TEM; (**c**) HRTEM image (with SAED pattern, inset); (**d**) XRD pattern of PdS_2_@GO


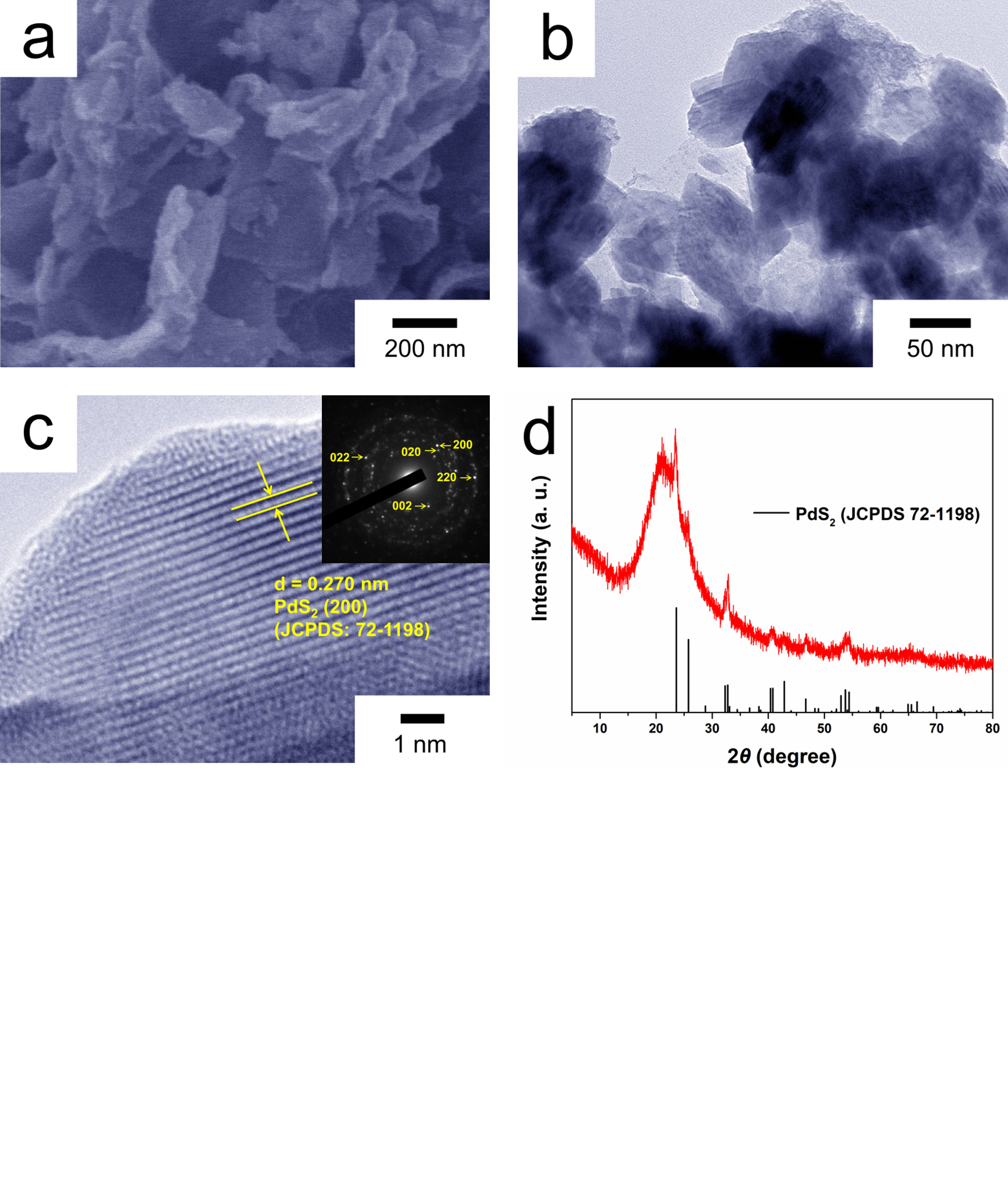


**Fig. S7** (**a**) SEM; (**b**) TEM; (**c**) HRTEM image (with SAED pattern, inset); (**d**) XRD pattern of PdS_2_@PPy/GO


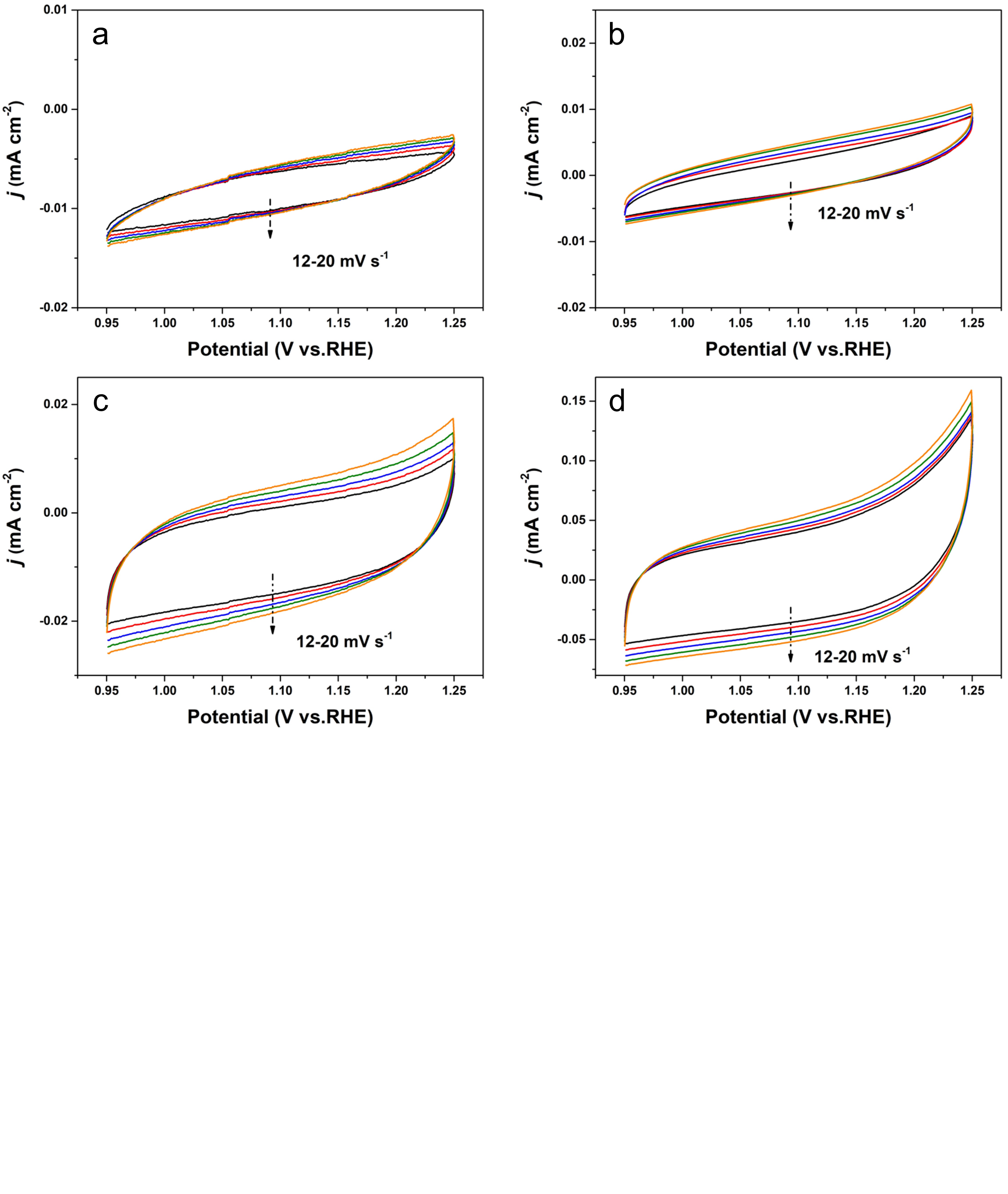


**Fig. S8** Cyclic voltammetry profiles of (**a**) PdS_2_, (**b**) PdS_2_@GO, (**c**) PdS_2_@PPy/GO and (**d**) PdS_2_@PVEIB/PPy/GO at the sweep rates


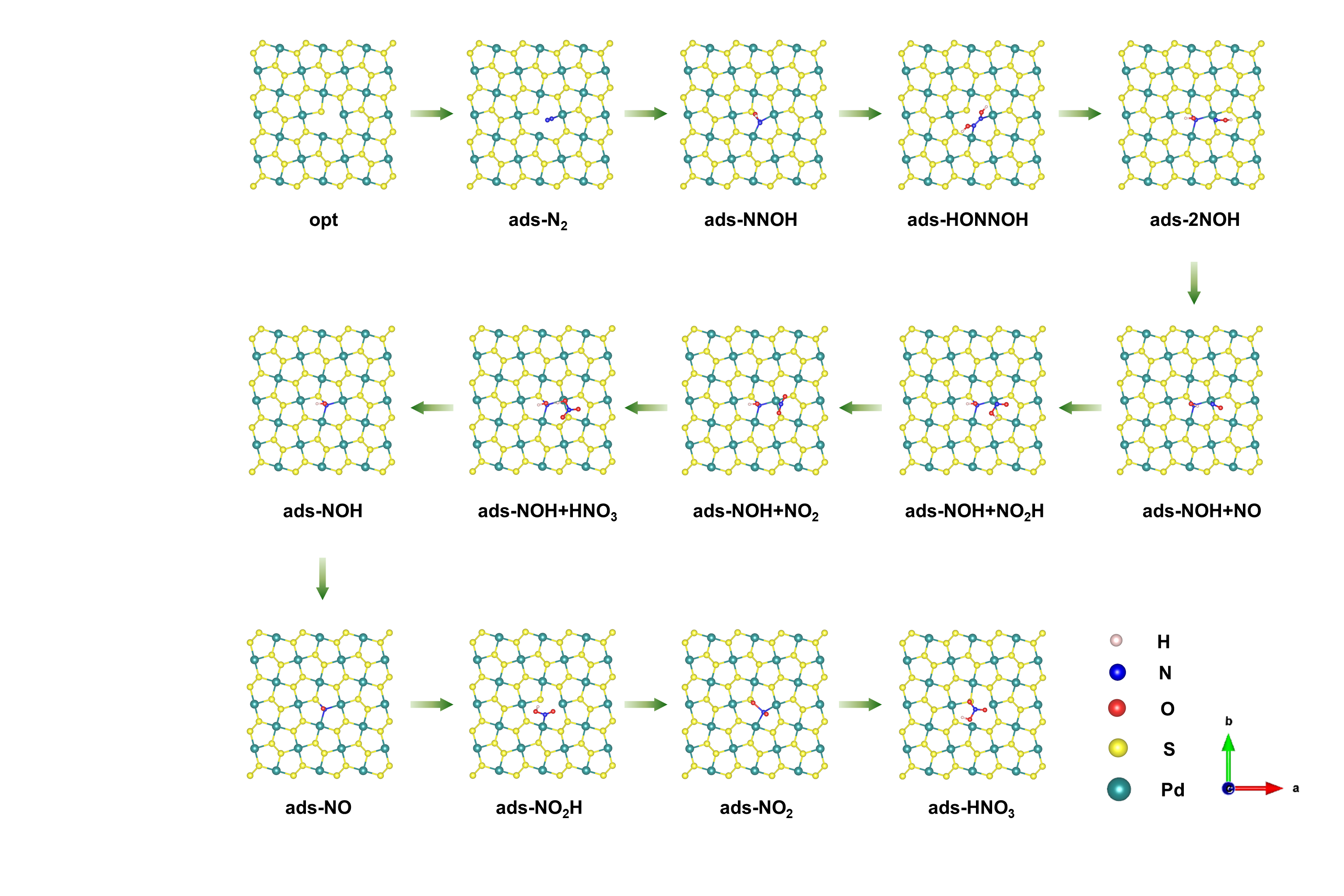


**Fig. S9** The atomistic structure scheme for describing the reaction pathway of N_2_ oxidation on the S-vacancy of PdS_2_ (002)

**
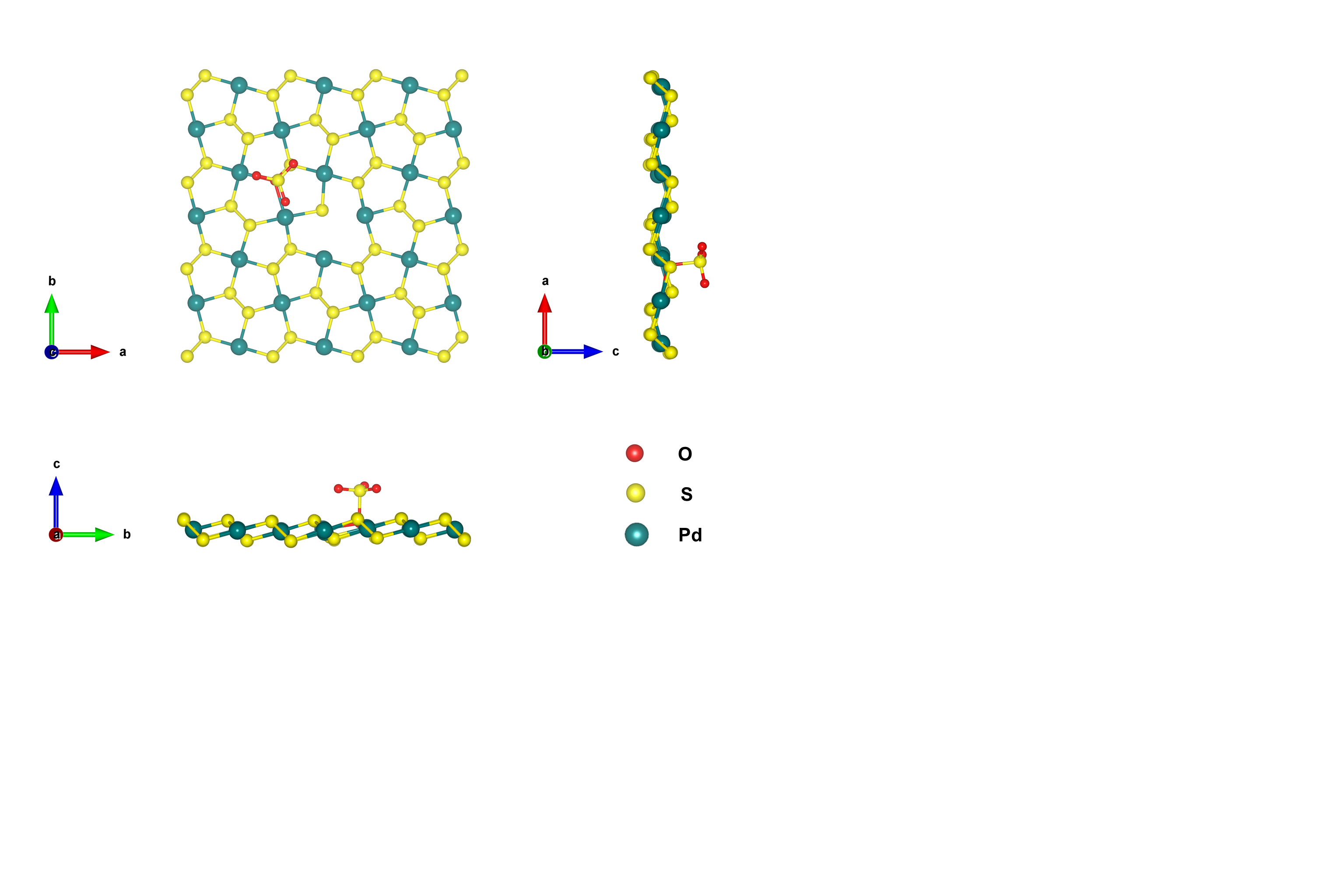
**

**Fig. S10** Top and side views of the in situ generated SO_4_^2-^ on a (002) surface of PdS_2_ monolayer with an S-vacancy

**Table S1** Different electrocatalysts synthesized by different supporters with their Pd content determined by ICP and calculated PdS_2_ loading content

| **Label** | **Electrocatalyst** | **Pd content by ICP (wt%)** | **PdS_2_ loading (wt%)** |
| --- | --- | --- | --- |
| a | GO | - | - |
| b | PPy/GO | - | - |
| c | PVEIB/PPy/GO |  |  |
| d | PdS_2_ | - | 100.00 |
| e | PdS_2_@GO | 24.17 | 38.76 |
| f | PdS_2_@PPy/GO | 20.74 | 33.26 |
| g | PdS_2_@PVEIB/PPy/GO | 22.14 | 35.51 |

**Table S2** The NOR performance comparison of PdS_2_@PVEIB/PPy/GO with the well-developed electrocatalysts for the NOR

| **Electrocatalyst** | **Electrolyte** | **Potential (vs. RHE)** | **FE (%)** | **NO_3_^-^ yield (μg h^-1^ mg_act._^-1^)** | **Stability** | **References** |
| --- | --- | --- | --- | --- | --- | --- |
| **PdS_2_@PVEIB/PPy/GO** | **0.1 M KOH** | **2.05** | **7.36** | **93.91** | **12** | **This work** |
| Pd-s PNSs | 0.1 M KOH | 1.75 | 2.5 | 18.6 | 72 | [S14] |
| ZnFeCoO_4_ | 1 M KOH | 1.6 | 10.1 | 8.06 | 24 | [S15] |
| Ru/TiO_2_ | 0.1 M Na_2_SO_4_ | 1.8 | 26.1 | 9.982 | 10 | [S16] |
| Pd_0.9_Ru_0.1_ | 0.1 M KOH | 1.7 | 0.61 | 4.836 | 5 | [S17] |
| Fe-SnO_2_ | 0.05 M H_2_SO_4_ | 1.96 | 0.84 | 42.904 | 10 | [S18] |
| Rh nanoparticles | 0.1 M KOH+0.5 M SO_4_^2-^ | 1.9 | - | 10.416 | 50 | [S19] |
| Ru-Mn_3_O_4_ | 0.1 M Na_2_SO_4_ | 2 | 6.33 | 35.34 | 10 | [S20] |
| Nanoporous B_13_C_2_ | 0.1 M Na_2_SO_4_ | 2.4 | 4.17 | 165.8 | - | [S21] |
| OPA-PCN-222(Fe) | 0.1 M HCl | 1.6 | 70.7 | 110.9 | 10 | [S22] |
| Pd-MXene | 0.01 M Na_2_SO_4_ | 0.4 | 11.34 | 2.8 | - | [S23] |
| AD-Fe NS | 0.05 M K_2_SO_4_ | 2.1 | 35.63 | 379.44 | 10 | [S24] |
| Pd/S-TiO_2_@2-MeIm/PPy/GO | 0.1 M KOH | 2.04 | 8.92 | 72.69 | 12 | [S25] |
| FeS_2_-TiO_2_@2-MeIm/PPy/GO | 0.1 M KOH | 2.04 | 4.38 | 137.04 | 12 | [S26] |
| D-CeO_2_/MQDs | 0.1 M KOH | 1.7 | 31.80 | 71.25 | 10 | [S27] |
| Sr_0.9_RuO_3_ | 0.1 M Na_2_SO_4_ | 2.2 | 38.6 | 1109.8 | 30 | [S28] |

**Table S3** The binding energies and assignments for C 1s, N 1s, O 1s, Pd 3d and S 2p in the XPS fitting spectra of PdS_2_@PVEIB/PPy/GO before and after NOR

| **Element** | **B. E. (eV) before NOR** | **B. E. (eV) after NOR** | **Attribution** | **Ref.** |
| --- | --- | --- | --- | --- |
| C 1s | 284.0 | 284.0 | sp^2^-hybridized carbon | [S29] |
|  | 284.7 | 284.7 | C-C/C-H, | [S30] |
|  | 285.5 | 285.5 | C-N/C=N of Py rings | [S31] |
|  | 286.5 | 286.5 | C-N^+^ of Py | [S32] |
|  | 287.5 | 287.5 | C-N^+^ of imidazolium ring | [S26] |
|  | 288.8 | 288.8 | O-C=O | [S33] |
|  |  | 291.8 | CF_2_ groups in Nafion | [S34] |
|  |  | 293.5 | CF_3_ groups in Nafion | [S35] |
|  |  | 292.6 | K^+^ 2p_3/2_ | [S36] |
|  |  | 295.7 | K^+^ 2p_1/2_ | [S37] |
| N 1s | 398.1 | 398.1 | pyridinic-N | [S38] |
|  | 400.1 | 400.1 | pyrrolic-N | [S39] |
|  | 401.5 | 401.5 | N^+^ in the imidazolium ring | [S40] |
| O 1s |  | 530.7 | OH^-^ | [S41] |
|  | 531.5 | 531.5 | C=O | [S42] |
|  | 532.3 | 532.3 | Pd 3p_3/2_ | [S43] |
|  | 533.2 | 533.2 | O−C=O in PPy/GO | [S44] |
|  | 534.1 | 534.1 | HO-C=O/C-OH | [S45, S46] |
|  | 535.6 | 535.6 | -SO_3_^-^/SO_4_^2-^ | [S47] |
| Pd 3d | 336.9 | 336.8 | Pd 3d_5/2_ of Pd-S bonding mode | [S48] |
|  | 342.1 | 342.0 | Pd 3d_3/2_ of Pd-S bonding mode | [S48] |
|  | 337.9 | 337.9 | Pd 3d_5/2_ of Pd-O bonding mode | [S49] |
|  | 343.1 | 343.1 | Pd 3d_3/2_ of Pd-O bonding mode | [S49] |
| S 2p | 161.5 | 161.4 | terminal S | [S50] |
|  | 162.4 | 162.3 | S 2p_3/2_ of Pd-S bonding mode | [S51] |
|  | 163.0 | 162.9 | S 2p_1/2_ of Pd-S bonding mode | [S51] |
|  | 163.6 | 163.5 | S 2p_3/2_ of bridging S bonding mode | [S52] |
|  | 164.4 | 164.3 | S 2p_1/2_ of bridging S bonding mode | [S53] |
|  | 166.0 | 166.0 | S-O | [S54] |
|  | 168.6 | 168.6 | SO_4_^2-^ | [S55] |

**Supplementary References**

1. W.S. Hummers Jr, R.E. Offeman, Preparation of graphitic oxide. J. Am. Chem. Soc. **80**(6), 1339 (1958). <https://doi.org/10.1021/ja01539a017>
2. P. Wang, Y. Zheng, B. Li, Preparation and electrochemical properties of polypyrrole/graphite oxide composites with various feed ratios of pyrrole to graphite oxide. Synth. Met. **166**, 33–39 (2013). <https://doi.org/10.1016/j.synthmet.2013.01.002>
3. H. Mao, X. Guo, Q. Fan, Y. Fu, H. Yang et al., Improved hydrogen evolution activity by unique NiS_2_-MoS_2_ heterostructures with misfit lattices supported on poly(ionic liquid)s functionalized polypyrrole/graphene oxide nanosheets. Chem. Eng. J. **404**, 126253 (2021). <https://doi.org/10.1016/j.cej.2020.126253>
4. H. Mao, M. Liu, Z. Cao, C. Ji, Y. Sun et al., Poly(4-vinylphenylboronic acid) functionalized polypyrrole/graphene oxide nanosheets for simultaneous electrochemical determination of catechol and hydroquinone. Appl. Surf. Sci. **420**, 594–605 (2017). <https://doi.org/10.1016/j.apsusc.2017.05.188>
5. R. Marcilla, J. Alberto Blazquez, J. Rodriguez, J.A. Pomposo, D. Mecerreyes, Tuning the solubility of polymerized ionic liquids by simple anion-exchange reactions. J. Polym. Sci. Part A Polym. Chem. **42**(1), 208–212 (2004). <https://doi.org/10.1002/pola.11015>
6. G. Kresse, J. Furthmüller, Efficiency of ab-initio total energy calculations for metals and semiconductors using a plane-wave basis set. Comput. Mater. Sci. **6**(1), 15–50 (1996). <https://doi.org/10.1016/0927-0256(96)00008-0>
7. G. Kresse, J. Furthmüller, Efficient iterative schemes for *ab initio* total-energy calculations using a plane-wave basis set. Phys. Rev. B Condens. Matter **54**(16), 11169–11186 (1996). <https://doi.org/10.1103/physrevb.54.11169>
8. J.P. Perdew, K. Burke, M. Ernzerhof, Generalized gradient approximation made simple. Phys. Rev. Lett. **77**(18), 3865–3868 (1996). <https://doi.org/10.1103/PhysRevLett.77.3865>
9. G. Kresse, D. Joubert, From ultrasoft pseudopotentials to the projector augmented-wave method. Phys. Rev. B **59**(3), 1758–1775 (1999). <https://doi.org/10.1103/physrevb.59.1758>
10. P.E. Blöchl, Projector augmented-wave method. Phys. Rev. B **50**(24), 17953–17979 (1994). <https://doi.org/10.1103/physrevb.50.17953>
11. H.J. Monkhorst, J.D. Pack, Special points for Brillouin-zone integrations. Phys. Rev. B **13**(12), 5188–5192 (1976). <https://doi.org/10.1103/physrevb.13.5188>
12. S. Grimme, J. Antony, S. Ehrlich, H. Krieg, A consistent and accurate *ab initio* parametrization of density functional dispersion correction (DFT-D) for the 94 elements H-Pu. J. Chem. Phys. **132**(15), 154104 (2010). <https://doi.org/10.1063/1.3382344>
13. S. Grimme, S. Ehrlich, L. Goerigk, Effect of the damping function in dispersion corrected density functional theory. J. Comput. Chem. **32**(7), 1456–1465 (2011). <https://doi.org/10.1002/jcc.21759>
14. S. Han, C. Wang, Y. Wang, Y. Yu, B. Zhang, Electrosynthesis of nitrate *via* the oxidation of nitrogen on tensile-strained palladium porous nanosheets. Angew. Chem. Int. Ed. **60**(9), 4474–4478 (2021). <https://doi.org/10.1002/anie.202014017>
15. C. Dai, Y. Sun, G. Chen, A.C. Fisher, Z.J. Xu, Electrochemical oxidation of nitrogen towards direct nitrate production on spinel oxides. Angew. Chem. Int. Ed. **59**(24), 9418–9422 (2020). <https://doi.org/10.1002/anie.202002923>
16. M. Kuang, Y. Wang, W. Fang, H. Tan, M. Chen et al., Efficient nitrate synthesis *via* ambient nitrogen oxidation with Ru-doped TiO_2_/RuO_2_ electrocatalysts. Adv. Mater. **32**(26), 2002189 (2020). <https://doi.org/10.1002/adma.202002189>
17. T. Li, S. Han, C. Wang, Y. Huang, Y. Wang et al., Ru-doped Pd nanoparticles for nitrogen electrooxidation to nitrate. ACS Catal. **11**(22), 14032–14037 (2021). <https://doi.org/10.1021/acscatal.1c04360>
18. L. Zhang, M. Cong, X. Ding, Y. Jin, F. Xu et al., A Janus Fe-SnO_2_ catalyst that enables bifunctional electrochemical nitrogen fixation. Angew. Chem. Int. Ed **59**(27), 10888–10893 (2020). <https://doi.org/10.1002/anie.202003518>
19. T. Li, S. Han, C. Cheng, Y. Wang, X. Du et al., Sulfate-enabled nitrate synthesis from nitrogen electrooxidation on a rhodium electrocatalyst. Angew. Chem. Int. Ed **61**(26), e202204541 (2022). <https://doi.org/10.1002/anie.202204541>
20. Z. Nie, L. Zhang, X. Ding, M. Cong, F. Xu et al., Catalytic kinetics regulation for enhanced electrochemical nitrogen oxidation by Ru-nanoclusters-coupled Mn_3_O_4_ catalysts decorated with atomically dispersed Ru atoms. Adv. Mater. **34**(14), 2108180 (2022). <https://doi.org/10.1002/adma.202108180>
21. J. Lan, M. Luo, J. Han, M. Peng, H. Duan et al., Nanoporous B^13^C2 towards highly efficient electrochemical nitrogen fixation. Small **17**(39), 2102814 (2021). <https://doi.org/10.1002/smll.202102814>
22. H. He, H.-K. Li, Q.-Q. Zhu, C.-P. Li, Z. Zhang et al., Hydrophobicity modulation on a ferriporphyrin-based metal–organic framework for enhanced ambient electrocatalytic nitrogen fixation. Appl. Catal. B Environ. **316**, 121673 (2022). <https://doi.org/10.1016/j.apcatb.2022.121673>
23. W. Fang, C. Du, M. Kuang, M. Chen, W. Huang et al., Boosting efficient ambient nitrogen oxidation by a well-dispersed Pd on MXene electrocatalyst. Chem. Commun. **56**(43), 5779–5782 (2020). <https://doi.org/10.1039/D0CC01759K>
24. Y. Guo, S. Zhang, R. Zhang, D. Wang, D. Zhu et al., Electrochemical nitrate production *via* nitrogen oxidation with atomically dispersed Fe on N-doped carbon nanosheets. ACS Nano **16**(1), 655–663 (2022). <https://doi.org/10.1021/acsnano.1c08109>
25. H. Mao, Y. Sun, H. Li, S. Wu, D. Liu et al., Synergy of Pd^2+^/S2−-doped TiO_2_ supported on 2-methylimidazolium-functionalized polypyrrole/graphene oxide for enhanced nitrogen electrooxidation. Adv. Mater. **36**(16), 2313155 (2024). <https://doi.org/10.1002/adma.202313155>
26. H. Mao, H. Li, Y. Sun, S. Wu, Q. Wu et al., Electrosynthesis of nitrate by FeS_2_-TiO_2_ heterogeneous nanoparticles supported on 2-methylimidazolium functionalized polypyrrole/graphene oxide. Chem. Eng. J. **489**, 151414 (2024). <https://doi.org/10.1016/j.cej.2024.151414>
27. Q. Li, D. Shen, Z. Xiao, X. Liu, X. Xu et al., Dual-shelled CeO_2_ hollow spheres decorated with MXene quantum dots for efficient electrocatalytic nitrogen oxidation. Small **21**(11), 2411665 (2025). <https://doi.org/10.1002/smll.202411665>
28. H. Zheng, Z. Ma, Y. Liu, Y. Zhang, J. Ye et al., Perovskite oxide as a new platform for efficient electrocatalytic nitrogen oxidation. Angew. Chem. Int. Ed. **63**(1), e202316097 (2024). <https://doi.org/10.1002/anie.202316097>
29. R. Hu, T. Furukawa, X. Wang, M. Nagatsu, Morphological study of graphite-encapsulated iron composite nanoparticles fabricated by a one-step arc discharge method. Appl. Surf. Sci. **416**, 731–741 (2017). <https://doi.org/10.1016/j.apsusc.2017.04.231>
30. H. Zhou, Y. Zhou, Y. Cao, Z. Wang, J. Wang et al., Hollow LDH cage covering with ultra-thin MXenes veil: Integrated micro-nano structure upon heat release suppression and toxic effluents elimination for polymer. Chem. Eng. J. **461**, 142035 (2023). <https://doi.org/10.1016/j.cej.2023.142035>
31. Y. Chen, S. Lyu, S. Han, Z. Chen, W. Wang et al., Nanocellulose/polypyrrole aerogel electrodes with higher conductivity *via* adding vapor grown nano-carbon fibers as conducting networks for supercapacitor application. RSC Adv. **8**(70), 39918–39928 (2018). <https://doi.org/10.1039/C8RA07054G>
32. M. Bharti, A. Singh, S. Samanta, A.K. Debnath, D.K. Aswal et al., Flexo-green Polypyrrole–Silver nanocomposite films for thermoelectric power generation. Energy Convers. Manag. **144**, 143–152 (2017). <https://doi.org/10.1016/j.enconman.2017.04.022>
33. H. Zhang, T. Wang, W.-Y. Chen, Y. Zhang, B. Sun et al., Derivation of oxygen-containing functional groups on biochar under non-oxygen plasma for mercury removal. Fuel **275**, 117879 (2020). <https://doi.org/10.1016/j.fuel.2020.117879>
34. C.E. Hamilton, J.R. Lomeda, Z. Sun, J.M. Tour, A.R. Barron, Radical addition of perfluorinated alkyl iodides to multi-layered graphene and single-walled carbon nanotubes. Nano Res. **3**(2), 138–145 (2010). <https://doi.org/10.1007/s12274-010-1007-3>
35. Y. Cai, J. Li, L. Yi, X. Yan, J. Li, Fabricating superhydrophobic and oleophobic surface with silica nanoparticles modified by silanes and environment-friendly fluorinated chemicals. Appl. Surf. Sci. **450**, 102–111 (2018). <https://doi.org/10.1016/j.apsusc.2018.04.186>
36. G. Yu, J. Wang, H. Ma, X. Liu, S. Qin et al., Exploring abundantly synergic effects of K-Cu supported paper catalysts using TiO_2_-ZrO_2_ mesoporous fibers as matrix towards soot efficient oxidation. Chem. Eng. J. **417**, 128111 (2021). <https://doi.org/10.1016/j.cej.2020.128111>
37. H. Che, J. Wang, X. Gao, J. Chen, P. Wang et al., Regulating directional transfer of electrons on polymeric g-C3N5 for highly efficient photocatalytic H_2_O_2_ production. J. Colloid Interface Sci. **627**, 739–748 (2022). <https://doi.org/10.1016/j.jcis.2022.07.080>
38. K. Zhang, Y. Xu, F. Liu, G.-P. Yan, S.-W. Guo, Preparation and properties of ZnS–CdSe@Co/N–C core/shell composites for visible light photoconversion of CO_2_. New J. Chem. **47**(27), 12550–12553 (2023). <https://doi.org/10.1039/D3NJ00827D>
39. Q. Li, D. Xu, J. Guo, X. Ou, F. Yan, Protonated g-C_3_N_4_@polypyrrole derived N-doped porous carbon for supercapacitors and oxygen electrocatalysis. Carbon **124**, 599–610 (2017). <https://doi.org/10.1016/j.carbon.2017.09.029>
40. S.K. Das, M.-O. Winghart, P. Han, D. Rana, Z.-Y. Zhang et al., Electronic fingerprint of the protonated imidazole dimer probed by X-ray absorption spectroscopy. J. Phys. Chem. Lett. **15**(5), 1264–1272 (2024). <https://doi.org/10.1021/acs.jpclett.3c03576>
41. D. Wang, W. Gong, J. Zhang, G. Wang, H. Zhang et al., *In situ* growth of MOFs on Ni(OH)_2_ for efficient electrocatalytic oxidation of 5-hydroxymethylfurfural. Chem. Commun. **57**(86), 11358–11361 (2021). <https://doi.org/10.1039/D1CC04680B>
42. Q. Lou, X. Yang, K. Liu, Z. Ding, J. Qin et al., Pressure-induced photoluminescence enhancement and ambient retention in confined carbon dots. Nano Res. **15**(3), 2545–2551 (2022). <https://doi.org/10.1007/s12274-021-3736-x>
43. R. Nandan, K.K. Nanda, A unique approach to designing resilient bi-functional nano-electrocatalysts based on ultrafine bimetallic nanoparticles dispersed in carbon nanospheres. J. Mater. Chem. A **5**(21), 10544–10553 (2017). <https://doi.org/10.1039/C7TA02293J>
44. J. Zhao, B. Wang, Q. Zhou, H. Wang, X. Li et al., Efficient electrohydrogenation of N_2_ to NH_3_ by oxidized carbon nanotubes under ambient conditions. Chem. Commun. **55**(34), 4997–5000 (2019). <https://doi.org/10.1039/C9CC00726A>
45. P. Song, L. Zhu, L. Wang, S. Song, L. Han, Nitrogen and oxygen Co-doped porous biocarbon@sulfur/carbon nanotubes as cathodes for high-performance lithium-sulfur batteries. Mater. Lett. **355**, 135539 (2024). <https://doi.org/10.1016/j.matlet.2023.135539>
46. S. Chen, T. Luo, K. Chen, Y. Lin, J. Fu et al., Chemical identification of catalytically active sites on oxygen-doped carbon nanosheet to decipher the high activity for electro-synthesis hydrogen peroxide. Angew. Chem. Int. Ed. **60**(30), 16607–16614 (2021). <https://doi.org/10.1002/anie.202104480>
47. Z. Tan, X. Qin, P. Cao, S. Chen, H. Yu et al., Enhanced electrochemical-activation of H_2_O_2_ to produce •OH by regulating the adsorption of H_2_O_2_ on nitrogen-doped porous carbon for organic pollutants removal. J. Hazard. Mater. **458**, 131925 (2023). <https://doi.org/10.1016/j.jhazmat.2023.131925>
48. A. Taherkhani, S.Z. Mortazavi, S. Ahmadi, A. Reyhani, Synchrotron EXAFS spectroscopy and density functional theory studies of Pd/MoS_2_ Schottky-type nano/hetero-junctions. Appl. Surf. Sci. **604**, 154557 (2022). <https://doi.org/10.1016/j.apsusc.2022.154557>
49. J. Zhang, J. Chen, Z. Li, H. Weng, Y. Xie et al., CO oxidation over PdOσ/Fe_1-_*_x_*WxO*_y_* catalysts and their SO_2_ resistance at relatively low temperature. Fuel **350**, 128802 (2023). <https://doi.org/10.1016/j.fuel.2023.128802>
50. H. Wang, G. Yan, M. Li, H. Ji, Y. Feng et al., Nucleophilic ring-opening of thiolactones: a facile method for sulfhydrylization of a carbon nanotube-based cathode toward high-performance Li–S batteries. ACS Sustainable Chem. Eng. **10**(15), 5005–5014 (2022). <https://doi.org/10.1021/acssuschemeng.2c00204>
51. F. Luo, P. Yu, J. Xiang, J. Jiang, S. Chen, *In situ* generation of oxyanions-decorated cobalt(nickel) oxyhydroxide catalyst with high corrosion resistance for stable and efficient seawater oxidation. J. Energy Chem. **94**, 508–516 (2024). <https://doi.org/10.1016/j.jechem.2024.03.006>
52. G. Liu, L. Ding, Y. Meng, A. Ali, G. Zuo et al., A review on ultra-small undoped MoS_2_ as advanced catalysts for renewable fuel production. Carbon Energy **6**(2), e521 (2024). <https://doi.org/10.1002/cey2.521>
53. S. Liu, X. Zhang, S. Wu, X. Chen, X. Yang et al., Crepe cake structured layered double hydroxide/sulfur/graphene as a positive electrode material for Li-S batteries. ACS Nano **14**(7), 8220–8231 (2020). <https://doi.org/10.1021/acsnano.0c01694>
54. R. Li, J. Li, K. Qi, X. Ge, Q. Zhang et al., One-step synthesis of 3D sulfur/nitrogen dual-doped graphene supported nano silicon as anode for Li-ion batteries. Appl. Surf. Sci. **433**, 367–373 (2018). <https://doi.org/10.1016/j.apsusc.2017.10.061>
55. M. Xiang, L. He, Q. Su, B. Sun, N. Wang et al., Supercapacitor properties of N/S/O Co-doped and hydrothermally sculpted porous carbon cloth in pH-universal aqueous electrolytes: Mechanism of performance enhancement. Chem. Eng. J. **485**, 149835 (2024). <https://doi.org/10.1016/j.cej.2024.149835>
